# Sinks as limited resources? A new indicator for evaluating anthropogenic material flows

**DOI:** 10.1016/j.ecolind.2014.06.027

**Published:** 2014-11

**Authors:** Ulrich Kral, Paul H. Brunner, Pi-Cheng Chen, Sih-Rong Chen

**Affiliations:** a Vienna University of Technology, Institute for Water Quality, Resource and Waste Management, Karlsplatz 13/226, A-1040 Vienna, Austria^1^; b National Taiwan University, Graduate Institute of Environmental Engineering, 71 Chou-Shan Road, Taipei 106, Taiwan

**Keywords:** Sink, Substance flow analysis, Resource management, Environmental management, PFOS, Copper, Lead

## Abstract

Besides recyclables, the use of materials inevitably yields non-recyclable materials such as emissions and wastes for disposal. These flows must be directed to sinks in a way that no adverse effects arise for humans and the environment. The objective of this paper is to present a new indicator for the assessment of substance flows to sinks on a regional scale. The indicator quantifies the environmentally acceptable mass share of a substance in actual waste and emission flows, ranging from 0% as worst case to 100% as best case. This paper consists of three parts: first, the indicator is defined. Second, a methodology to determine the indicator score is presented, including (i) substance flows analysis and (ii) a distant-to-target approach based on an adaptation of the Ecological Scarcity Method 2006. Third, the metric developed is applied in three case studies including copper (Cu) and lead (Pb) in the city of Vienna, and perfluorooctane sulfonate (PFOS) in Switzerland. The following results were obtained: in Vienna, 99% of Cu flows to geogenic and anthropogenic sinks are acceptable when evaluated by the distant-to-target approach. However, the 0.7% of Cu entering urban soils and the 0.3% entering receiving waters are beyond the acceptable level. In the case of Pb, 92% of all flows into sinks prove to be acceptable, and 8% are disposed of in local landfills with limited capacity. For PFOS, 96% of all flows into sinks are acceptable. 4% cannot be evaluated due to a lack of normative criteria, despite posing a risk for human health and the environment. The examples demonstrate the need (i) for appropriate data of good quality to calculate the sink indicator and (ii) for standards, needed for the assessment of substance flows to urban soils and receiving waters. This study corroborates that the new indicator is well suited as a base for decisions regarding the control of hazardous substances in waste and environmental management.

## Highlights

• Sinks are required to accommodate wastes and emissions.
• Anthropogenic sinks are provided where capacities of geogenic sinks are lacking.
• A new indicator discriminates between acceptable and unacceptable flows to sinks.
• The indicator is applied in three case studies on urban and national scales.
• Implications for resource, waste and environmental management are discussed.

## Graphical abstract

**Figure:**
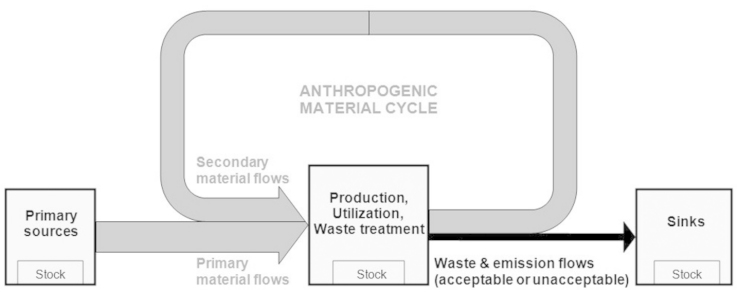

## 1 Introduction

“I do not worry about peak oil whatsoever. We have plenty of oil, gas, and coal to last for hundreds of years, and we are not running out. But we are running out of room in the atmosphere to store our exhaust.” Schnoor (2013) highlights the sink “atmosphere” as constraint for anthropogenic carbon before the sources run dry. The overriding question is if we are running out of “room in sinks” for other substances, too. Annually, millions of tons of materials are exploited from the earth crust or are produced synthetically, and processed into consumer and investment goods. After years or decades in use, the materials are discarded and meet their fate in terms of recycling or disposal in sinks. Therefore, geogenic sinks are available to a certain extent and anthropogenic sinks have to be provided where geogenic sinks are lacking. Geogenic sinks are part of biogeochemical cycles (e.g. Abeles et al., 1971, Berg and Dise, 2004, Feichter, 2008, Fong and Zedler, 2000, ICSU, 1989, Molina and Rowland, 1974, Paterson et al., 1996, Yanai et al., 2013). Anthropogenic sinks are manmade and refer to technologies such as incinerators, sanitary landfills, and sewage treatment plants (e.g. Brunner, 1999, Brunner and Tjell, 2012, ISWA, 2013, Morf and Brunner, 2005, Vogg, 2004, Zeschmar-Lahl, 2004). In general, materials must be directed to sinks in a way that no adverse effects arise for humans and the environment (Tarr, 1996).

To avoid unacceptable overloads, several authors have suggested metrics that focus on the relation between anthropogenic off-flows and potential impacts (Table 1). In common, these metrics (i) operate on a substance specific level, (ii) focus on human activities within regions, and (iii) work with a set of indicators. To calculate the indicator, a combination of descriptive and normative assessment methods is needed:

• Descriptive methods analyze the fate and behavior of substances through the anthroposphere and the environment. For this purpose, the tools substance flow analysis (SFA) and environmental fate modeling (EFM) have been developed (e.g. Brunner and Rechberger, 2004, Mackay et al., 2006, OECD, 2007, UNEP, 2002). To calculate *pressure indicators*, researchers devoted much effort to quantify substance flows from human activities into geogenic and anthropogenic sinks (e.g. Buser and Morf, 2009, Chen and Graedel, 2012, Henseler et al., 1992, Ott and Rechberger, 2012).
• Normative methods focus on the cause-effect chain of substances. Depending on the available knowledge, they either refer to “known damage due to known causalities”, or “known damages due to unknown causalities”, or “unknown damage due to unknown causalities” (adopted from Hofstetter, 1998). If damage and causalities are known, *impact indicators* can be provided. Therefore the tools risk assessment (RA) and life cycle impact assessment (LCIA) have been developed. LCIA focuses on the assessment of emissions along the whole life cycle chain of products and services rather than on emissions from entire human activities within regions (Loiseau et al., 2012). In general, LCIA methods rely on the scientific treatment of cause-effect relations from the intervention level toward the impact level. The LCIA method “Ecological Scarcity 2006” is an exception, because it considers the definition of critical flows into sinks based on legal limits and political agreements (Jungbluth et al., 2012). However, for the majority of substances placed on the market, the damages and causalities are partly or totally unknown (Berg and Scheringer, 1994, Grandjean, 2013). In this case, *proxy indicators* with more or less predictive power are used to approximate potential impacts.

**Table 1:** Selected studies applying pressure, proxy and impact oriented indicators characterizing environmental sustainability.

Reference	Spatial level	Pressure indicators^a^	Proxy indicators^b^	Impact indicators^c^
Alfsen and Sæbø (1993)	Norway		X	
Gilbert and Feenstra (1994)	Netherlands		X	
Nilsson and Bergström (1995)	Sewage Treatment Plant	X		
Azar et al. (1996)	World	X	X	
UNCSD (1996)	Not specified	X	X	X
Van der Voet (1996)	European Union	X	X	
Guinée et al. (1999)	Netherlands	X	X	X
Umweltbundesamt (1999)	Austria	X	X	
UNCHS (2001)	World	X		
Graymore et al. (2010)	World	X	X	
EEA (2012)	European Union	X	X	X

a Examples for pressure indicators are the amount of waste and emission flows.

b Examples for proxy indicators are (i) the spatial and temporal range of substances (Scheringer and Berg, 1994), (ii) the persistence, bio-accumulation, and toxicity of substances (European Parliament, 2006), (iii) legal limits or political agreements (Frischknecht et al., 2009), (iv) the ratio of anthropogenic to geogenic substance flows (Förstner and Müller, 1973; Reimann and de Caritat, 2005), and (v) exposure assessments (U.S. EPA, 2011).

c Examples for impact indicators are the number of human deaths due to certain substance flows into geogenic sinks.

Summarizing, the indicators developed so far focus on certain levels along the cause-effect chain. This includes the intervention level (*pressure indicators*), the effect level (*impact indicators*) or a level between intervention and effect (*proxy indicators* toward impacts). To our knowledge, individual indicators have not been linked yet systematically in view of ecological and human health assessment of regions. At present, the question “Which amounts of waste and emission flows are acceptable and unacceptable, respectively?” cannot be answered with a single indicator. To overcome this gap, Döberl and Brunner (2004) proposed to amend the tool box of sustainability metrics by the following indicator:

(1) $$\frac{\text{Amount}\;\text{of}\;\text{substances}\;\text{a}\;\text{region}\;\text{or}\;\text{process}\;\text{directs}\;\text{into}\;\text{appropriate}\;\text{final}\;\text{sinks}}{\text{Total}\;\text{amount}\;\text{of}\;\text{substances}\;\text{emitted}\;\text{by}\;\text{a}\;\text{region}\;\text{or}\;\text{process}}$$

Beyond the definition of the indicator, there is no operationalization in terms of assessment methods presented. However, the denominator of Eq. (1) refers to the intervention level and the numerator of Eq. (1) refers to a final level along the life cycle chain.

The present paper is inspired by Eq. (1), and advances it further to make it operational for application. The aim of the paper is to develop an assessment method that

• is able to consider specific substances,
• takes into account discarded material flows (wastes, emissions, substance flows from wear, corrosion, and weathering) from human activities within a spatial unit,
• covers geogenic and anthropogenic sinks for discarded material flows,
• allows the integration of normative criteria such as *proxy* and *impact criteria*,
• consists of a quantifiable indicator.

To achieve this goal, we relate acceptable to actual substance flows into sinks. Actual flows are determined by regional SFA, usually on an annual base. Acceptable flows can be determined by any environmental assessment method. We have chosen a distant-to-target approach according to the Ecological Scarcity (ES) method, and apply this framework in three case studies. The indicator score is determined for (1) copper (Cu) in the city of Vienna, (2) lead (Pb) in the city of Vienna, and (3) perfluorooctanesulfonate (PFOS) in Switzerland. Based on the findings, we present options to control the indicator score. The resulting indicator serves as a guide to identify potential constraints for sinks to accommodate waste and emission flows. The indicator is intended to support material management in view of potential sink limitation. Accordingly, we propose to add this indicator to existing metrics for characterizing the environmental dimension of sustainability.

## 2 Material and methods

In the following sections, we (i) define the indicator, (ii) present the methods for calculating the indicator score, and (iii) apply the metric in three case studies.

### 2.1 Indicator definition

The sink indicator (*λ*) quantifies the environmentally acceptable mass share of a substance in actual waste and emission flows. The score ranges between 0% and 100% and is displayed as in Fig. 5. The sink indicator is defined by

(2) $$\lambda =\frac{F_{\text{a}}}{F}*100$$

where *F*_a_ is the sum of acceptable flows in a region (see Eq. (6)) and *F* is the sum of actual flows in a region (see Eq. (3)).

(3) $$F=\sum_{i=1}^{n}F_{i}\;\text{with}$$

(4) $$F_{i}=\left\{\begin{array}{c} \beta_{i}\;\text{for}\;\beta_{i}>0\\ 0\;\text{for}\;\beta_{i}\leq 0 \end{array}\right)\;\text{with}$$

(5) $$\beta_{i}={\dot{m}}_{\text{in},i}-{\dot{m}}_{\text{out},i}$$

where *F*_*i*_ is an actual flow in a region and *i* is an index for a process. Hence, *β*_*i*_ is the net flow of a process, ${\dot{m}}_{\text{in},i}$ is the sum of flows into a process, and ${\dot{m}}_{\text{out},i}$ is the sum of flows out of a process (see Fig. 2). If *β*_*i*_ > 0, than *F*_*i*_ is equal to the positive net flow of a process. In this case, the net flow could either be a net addition to stock or the transformed mass share of the substance. The method for calculating the actual flows is presented in Section 2.2.2.

(6) $$F_{\text{a}}=\sum_{i=1}^{n}F_{\text{a},i}\;\text{with}$$

(7) $$F_{\text{a},i}=\left\{\begin{array}{c} F_{\text{c},i}\;\text{for}\;\alpha_{i}\geq 0\\ F_{i}\;\text{for}\;\alpha_{i}<0 \end{array}\right)\;\text{with}$$

(8) $$\alpha_{i}=F_{i}-F_{\text{c},i}$$

where *F*_a,*i*_ is an acceptable flow in a region, and *i* is an index for each processes. Hence, *α*_*i*_ is the distance-to-target value, *F*_*i*_ is the actual flow, and *F*_c,*i*_ is the critical flow. A critical flow represents *proxy criteria* such as political targets or *damage criteria* such as accepted human health risks. The method for calculating the critical flow is presented in Section 2.2.3.

**Fig. 1:**
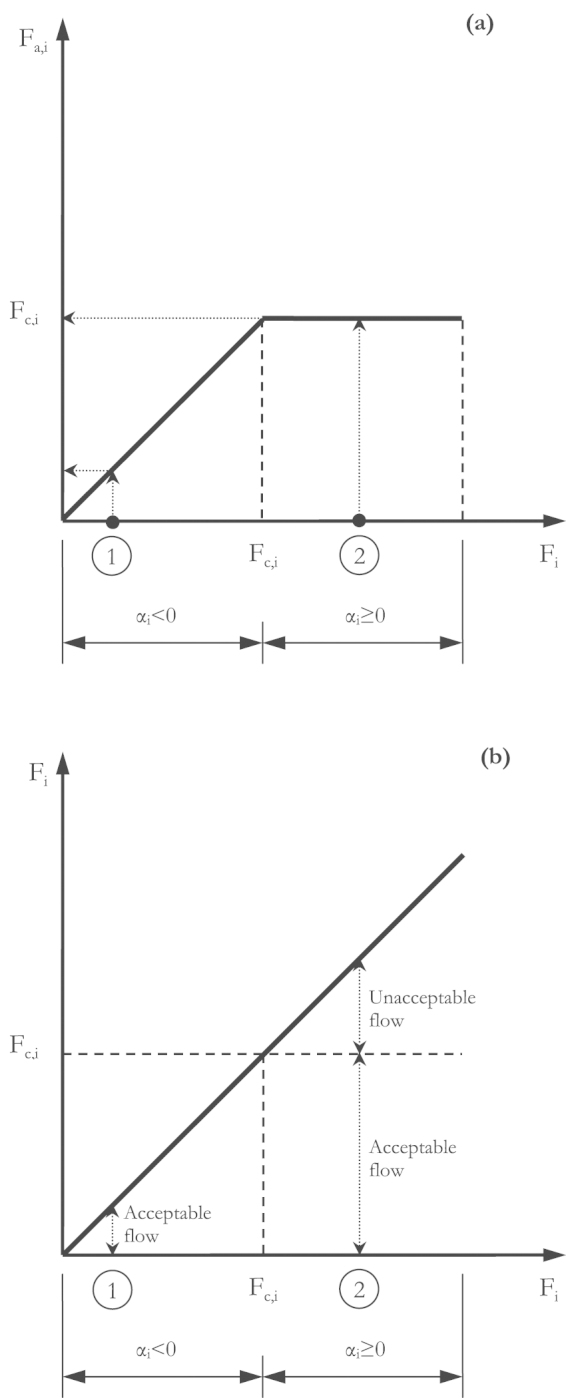
(a) Acceptable flow *F*_a,*i*_ as a function of the distant-to-target value *α_i_*. Example 1 demonstrates that for *α_i_* the acceptable flow *F*_a,*i*_ as equal to the actual flow *F_i_*. Example 2 demonstrates that for *α_i_* the acceptable flow *F*_a,*i*_ is equal to the critical flow *F*_c,*i*_. (b) The combination of the actual flow *F_i_* and the distant-to-target value *α_i_* yields two potential sub-flows: acceptable and unacceptable flows. Example 1 demonstrates for *α_i_* the results of an acceptable flow. Example 2 demonstrates for *α_i_* the result of acceptable and unacceptable flows.

Fig. 1(a) shows the acceptable flow *F*_a,*i*_ as a function of the distance-to-target value *α*_*i*_. If *α*_*i*_ < 0 then the actual flow *F*_*i*_ represents the acceptable flow *F*_a,*i*_. If *α*_*i*_ ≥ 0 then the critical flow *F*_c,*i*_ represents the acceptable flow *F*_a,*i*_. Fig. 1(b) plots the actual flow *F*_*i*_ on both axes in combination with the distant-to-target value *α*_*i*_. This produces three potential sub-flows: first, the acceptable flow is the mass flow below the actual flow (for *α*_*i*_ < 0) or below the critical flow (for *α*_*i*_ ≥ 0). Second, the unacceptable flow is the mass flow above the critical flow. Third, the tolerable flow is the mass flow below the critical flow and above the actual flow. In other words, the tolerable flow expresses the potential to increase the actual flow without violating normative criteria.

### 2.2 Methods for calculating the indicator score

To calculate the indicator score four steps are required (Fig. 3): first, the scope of assessment is defined. Second, a descriptive assessment of flows yields the sum of actual flows *F*. Third, a normative assessment of flows yields the sum of acceptable flows *F*_a_. Fourth, the indicator score is calculated.

#### 2.2.1 Scope

Setting the scope of the assessment includes the selection of a substance, a reference region and a period of interest. First, the notion “substance” is defined as “Matter of constant composition best characterized by the entities (atoms, molecules, formula units) it is composed of” (Nic et al., 2012). This is in line with the SFA framework used in this study, which defines the notion as “any (chemical) element or compound composed of uniform units” (Brunner and Rechberger, 2004). Second, the assessment deliberately focuses on regional flows instead of all flows along the life cycle chain of products. A region is bounded by administrative limits. Accordingly, communities, cities, federal states, nations or continents are subjects of the assessment. Third, setting the system boundary in time includes the selection of a reference period (day, week, month, year, decade, and so on) and a reference point in time (e.g. the year 2013). Temporal variations of flows within the reference period are often neglected because of lack of data.

#### 2.2.2 Descriptive assessment

**Fig. 2:**
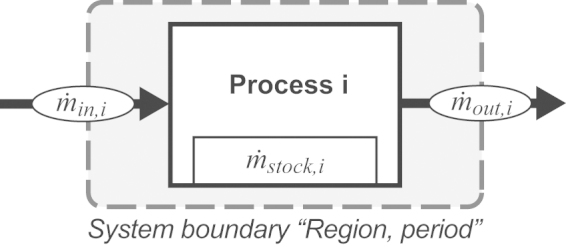
Plot of a generic process *i* where ${\dot{m}}_{\text{in},i}$ is the substance flow (mass per time) entering process *i*, ${\dot{m}}_{\text{out},i}$ is the substance flow (mass per time) leaving process *i* and ${\dot{m}}_{\text{stock},i}$ is the resulting alteration of mass (mass/time) within process *i*.

To quantify the sum of actual flows *F* (Eq. (3)), four steps are needed: first, to investigate into the anthropogenic metabolism, SFA has been proven to be a practical tool. It tracks the pathway of selected substances through systems such as households, enterprises, cities or regions. The applied methodology is in accordance with Baccini and Bader (1996) and Brunner and Rechberger (2004). The model development focuses on the identification of relevant processes and their links in terms of flows. Fig. 4 presents a framework for developing the SFA model. Model equations define the flows and stocks with the help of input parameters. Next, balance equations are applied for each process (Fig. 2, Fig. 4). The software STAN is used for data reconciliation and error propagation in order to balance mass flows and stocks (Cencic, 2012). Second, Sankey-Diagrams are elaborated to present SFA results (Schmidt, 2008). Third, the actual flows are determined. Therefore, the net flow *β*_*i*_ of each process (Eq. (5)) is calculated and displayed. This kind of plot allows the comparisons of various Sankey-Diagrams in a comparable manner. A positive net flow (*β*_*i*_ > 0) indicates a sink process, and a negative net flow (*β*_*i*_ < 0) indicates a source process. *F*_*i*_ The actual flow *F*_*i*_ is defined as a positive net flow into a sub-process within the process “Waste management” and “Environment” (Eq. (4)). Fourth, the actual flows are summed up (Eq. (3)).

**Fig. 3:**
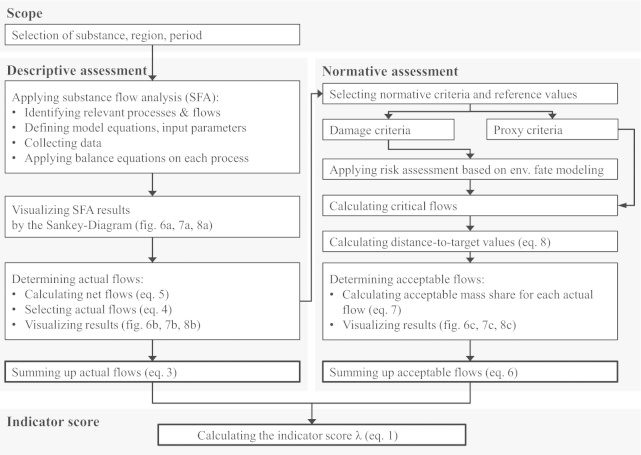
Framework to calculate the indicator score.

**Fig. 4:**
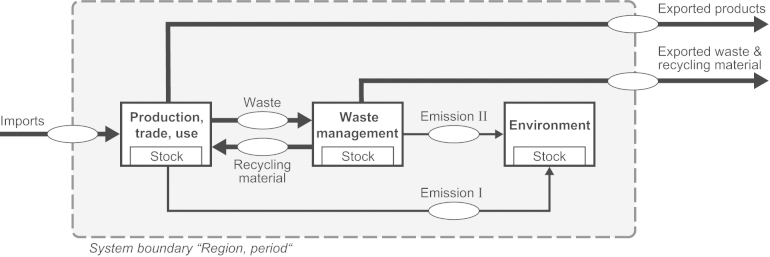
The SFA model refers to a specific substance, region and period of time. It includes processes in the, production, trade and use phase, in the waste management sector, and in the environment. Flows link the processes. The flows assessed by the indicator *λ* include the actual flows within the flows “Waste”, “Emissions I”, and “Emissions II”.

For two out of the three case studies, we use SFA data that have already been published: Cu in Vienna for the year 2008 (Kral et al., 2014) and PFOS in Switzerland for the year 2007 (Buser and Morf, 2009). The third case study focuses on Pb in Vienna for the year 2008. Background datasets can be found in the supplement information. In common, the Sankey-Diagram serves as starting point for calculating the net flows *β*_*i*_ and for filtering the actual flows *F*_*i*_.

#### 2.2.3 Normative assessment

To determine the acceptable flow *F*_a_ (Eq. (6)), four steps are needed: first, normative criteria and reference values define the acceptance of flows into sinks. Criteria and reference values are derived from goal oriented frameworks with respect to waste and emissions, such as regulations, standards, political agreements, or concepts for sustainable resource use like “clean cycles and final sinks” (Brunner, 2010, Kral et al., 2013), and “gradle to gradle” (Mulhall and Braungart, 2010). The definition of criteria depends on circumstances in the case study region. The circumstances might change over time, for example, as a consequence of new scientific knowledge, by improved data availability, and by changes in the ethical value-sphere. The circumstance might also vary from region to region, for example, as a consequence of initiatives to increase the recycling rate, different political agreements for accepted emission rates, and environmental quality standards. To select normative criteria and reference values in a specific region, the outcome of stakeholders’ involvement might be considered, without being further discussed in this article. To consider various criteria in the indicator framework, a criteria is related at any stage throughout the cause-effect chain of a substance, and a reference value determines the normative criteria in a quantitative manner. Table 2 gives some examples for *proxy criteria* at the stage of pressure, state, exposure and effect, and *damage criteria* at the stage of damage. Second, the critical flows are calculated based on models that establish causal links between the actual flow and the reference value attributed. Hence, the actual flow *F*_*i*_ is varied as long as the reference value is achieved. The resulting flow is called critical flow *F*_c,*i*_. In the case of multiple criteria for a single actual flow, the most stringent criteria is selected. Third, the distant-to-target value (Eq. (8)) determines the acceptable flow *F*_a,*i*_ (Eq. (7)). Fourth, the acceptable flows are summed up (Eq. (6)).

**Table 2:** Examples for normative criteria and reference values in context of the cause-effect chain, from the source toward the damage.

Stage	Stage description	Normative criteria	Reference values
Source	Potential of waste and emissions	n.r.	n.r.
Pressure	Flow into sink	Emission rates Waste into landfill	Legal limits, political agreements
State	Substance fate in (a) geogenic and (b) anthropogenic sinks.	Substance concentration, accumulation or transformation rate in (a) environmental media and (b) recycling goods, landfills, underground storage facilities.	Geogenic reference values in soil; approved landfill capacity and disposal time.
Exposure	Standard characteristics of exposed organism	Exposed dose, collective effective dose	MAK values, BAT values
Effect	Dose–response-relationship	Number and type of human diseases, number of vanishing plant species	Accepted number of cases regarding an human disease
Damage	Damage to human health or ecosystem quality	DALYs, QALYs, share of vanishing plant species per area and time unit.	Value weighted DALYs, accepted share of vanishing plant species per area and time unit

*Notes*: n.r.: not relevant; MAK values: maximum concentrations at the workplace; BAT values: Biological Tolerance Values; DALY: disability-adjusted life years or disease-adjusted life years; QALY: quality adjusted life year.

In view of the case studies, the selection of normative criteria and references values is based on *F*_c,*i*_ the Ecological Scarcity (ES) method (Frischknecht et al., 2009) including the following adoptions:

(1) ES method provides critical flow data for Switzerland. We adopt the data according to local circumstances in the case study region. For example, critical metal flows into surface waters refer to local environmental quality standards for surface waters of the specific case study country.
(2) ES method uses *proxy criteria* such as national reduction targets for greenhouse gas emissions and legal standards for heavy metal concentrations in surface waters. In addition, we introduce *impact criteria* such as human health risk. The integration of *impact criteria* demonstrates an additional option for critical flow determination. For human health risk, the cancer risk level (RL) and the non-cancer hazard-index (HI) represent two *impact criteria*.
(3) The ES method does not include PFOS as substance of interest. Legal limits in terms of concentrations in various emission flows have not been published yet. To assess flows into surface waters, we use an U.S. based reference concentration as *proxy criteria*.
(4) ES method assesses waste flows into landfills based on standards for the carbon content. We replace this *proxy criterion* with the constraints given by the official permission for each landfill. Therefore, the remaining landfill volume is divided by the approved, remaining time for disposal. The conversion of the annual volume flow into mass yields the annual critical flow into landfills.
(5) ES method aims at assessing impacts of waste and emission flows. Flows without impacts are not taken into account. But, they are of concern for the applied method. For example, organic substances are transformed mainly into carbon dioxide and water in incinerators, and are not present in their original form anymore. Accordingly, we introduce *proxy criteria* for organic substances in incineration. The critical flow is defined with the capacity of the incinerator in mass per year.

The applied method is in line with ES methodology as follows:

(1) Heavy metals to soils: The Swiss Regulation on the Impact on Soils (Schweizer Bundesrat, 1998) aims to ensure long-term soil fertility. Accumulation of heavy metals in soil is not accepted. ES method defines the critical flow as the heavy metal uptake through plants. This simplified approach neglecting leaching from the soil might be justified due to a lack of more precise regional data, but should be amended in the future.
(2) Hazardous waste to underground storage facilities: Switzerland has no appropriate storage facilities and thus exports hazardous waste to foreign underground storages facilities. In consultation with Swiss authorities, ES method sets the actual flow equal to the critical flow. Just as Switzerland, Austria exports waste into underground storage facilities. This justifies the same definition of the critical flow.

#### 2.2.4 Indicator score

**Fig. 5:**
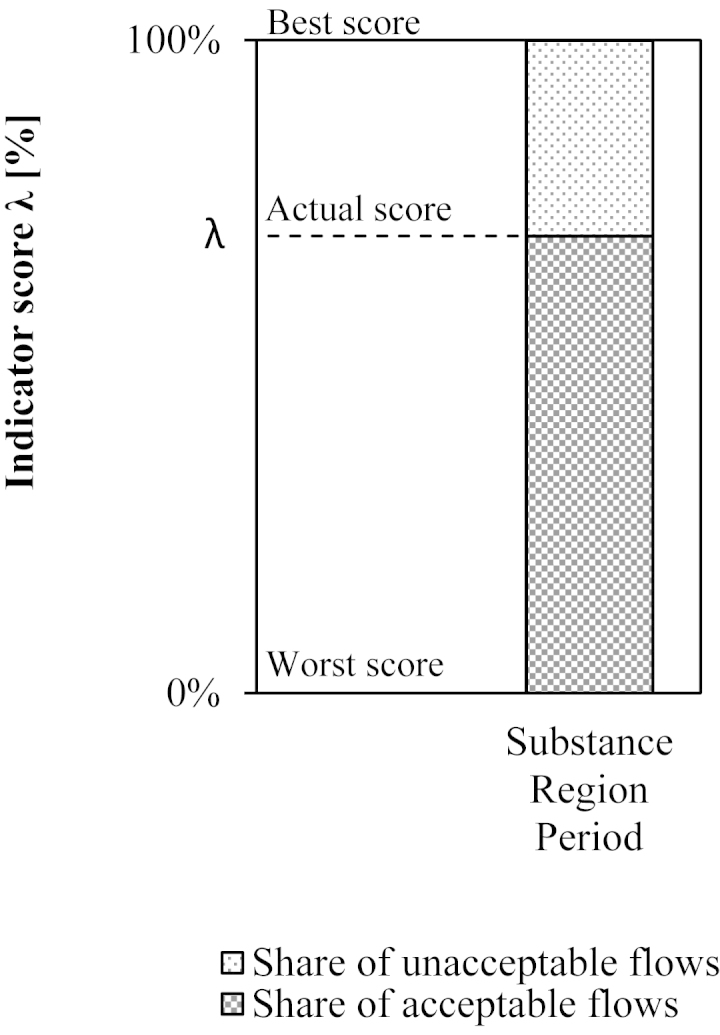
Generic plot of the indicator score *λ* shows both, the amount of acceptable and unacceptable flows into sinks. The score refers to the total flows into sinks of a given substance, within a given region and within a given time period.

Applying descriptive and normative assessment methods result in an indicator score, ranging from 0% to 100% (Fig. 5). The indicator takes 100% of actual flows into account, and discriminates between acceptable and unacceptable flows. Either all actual flows are fulfilling criteria of acceptability (*λ* = 100%), at least one flow is unacceptable (0% < *λ* < 100%), or all actual flows are unacceptable (*λ* = 0%). Accordingly, the positive connotation of the score can be seen as *the more the better*.

In general, the interpretation of the indicator score depends on the selection of (i) actual flows, (ii) normative criteria and references values, and (iii) data availability:

(i) The export flow “Exported waste & recycling material” crosses the spatial system boundary and enters external regions (see Fig. 4). It might be that the flow is not acceptable due to local circumstances in the export region, for instance, as a consequence of missing standards for pollution control, a lack of environmental sound treatment and recycling facilities, and sanitary landfills. The relevance of waste exports fraught with risk is well documented, for example, for waste electrical and electronic and equipment (e.g. Sthiannopkao and Wong, 2013, Widmer et al., 2005). In view of the present case studies, the flow “Exported waste & recycling material” is allocated to the external region and not taken into account, except those into underground storage farcicalities. To allocate flows into external sinks to the export region, critical flows for “Exported waste & recycling material” have to be defined.
(ii) Normative criteria and reference values can be derived from goal oriented frameworks such as regulations, standards, and political agreements. If the goals cannot be operationalized in terms of normative criteria and reference values, or if criteria are not considered in the indicator score calculation, the indicator score lacks of interpretational power regarding the goal oriented concepts. For example, conclusions regarding the effectiveness of recycling initiatives fail, if a normative criteria regarding recycling is missing.
(iii) Data acquisition is based on a bottom-up approach, supposing appropriate data quality and quantity. If data is lacking, the outcomes point to data requirements that have to be met before implications for environmental and waste management can be identified.

### 2.3 Case studies

The following three case studies are used to demonstrate the application of the sink indicator: Cu in Vienna, Pb in Vienna, and PFOS in Switzerland (Table 3). First, we highlight the motivation for the case study selection and briefly explain the background. Second, we present detailed Sankey-Diagrams including substance flows and calculate the sum of actual flows *F* according to Eq. (3). Third, we calculate the critical flows in order to discriminate between acceptable and unacceptable flows, respectively. The sum of acceptable flow yields *F*_a_ according to Eq. (6). Results are presented in Table 4.

**Table 3:** Case study overview.

Substance	Region	Year	Descriptive method	Number of actual flows	Number of sink processes	Type of criteria	SFA data reference
Cu	Vienna	2008	SFA	8	4	Proxy criteria	Kral et al. (2014)
Pb	Vienna	2008	SFA; EFM	9	7	Proxy and damage criteria	Supplement information
PFOS	Switzerland	2007	SFA	6	5	Proxy criteria	Buser and Morf (2009)

*Notes*: Cu: copper; Pb: lead; PFOS: perfluorooctanesulfonate; SFA: substance flow analysis; EFM: environmental fate modeling.

#### 2.3.1 Cu in Vienna

**Fig. 6:**
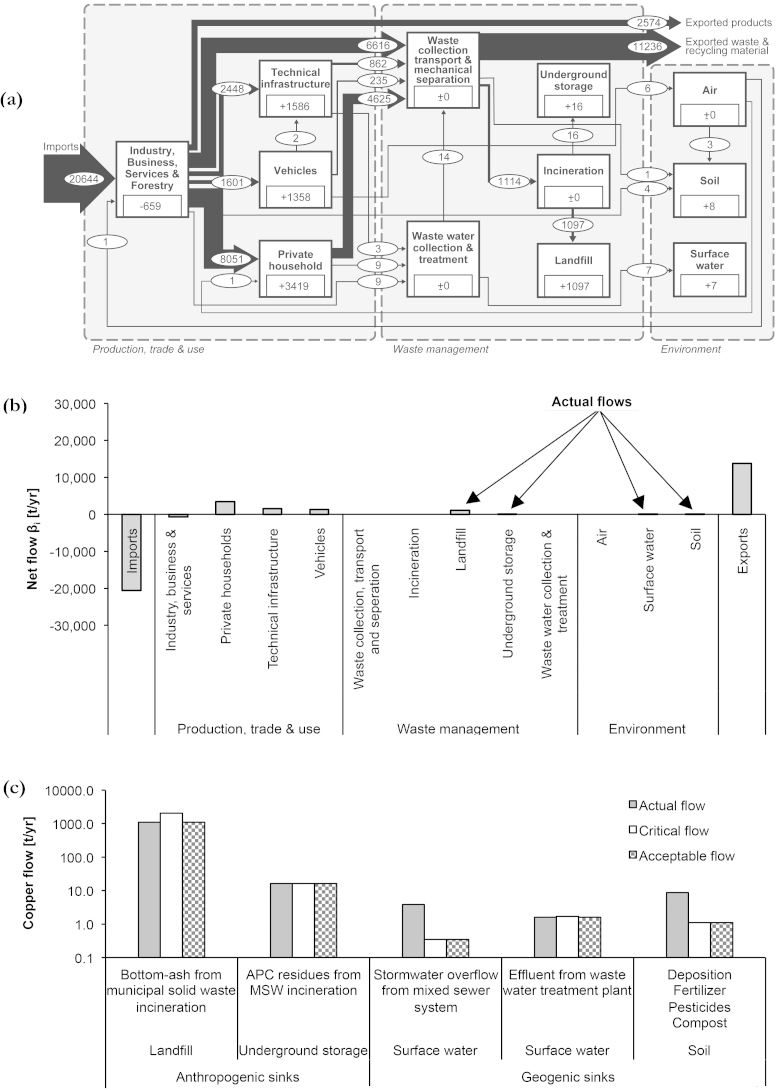
(a) Sankey-Diagram for copper in Vienna for the year 2008. Annual flow rates and changes in stocks are given in tons per year (t/yr), for stocks in tons (t). The flows are represented as Sankey diagrams proportional to the flow rate; figures for stocks are given within the process boxes. Deviations from mass balance are due to rounding. (b) The Source-Sink-Diagram presents the net flows *β_i_* for each process *i*. The actual flows are positive net flows into the waste management sector and the environment. (c) The plot shows the results from normative assessment, namely the rated flows into sinks.
(iii) To determine the critical flows, we use *proxy criteria.* The critical flow of bottom-ash into landfill results from the available landfill volume of 3.45 million cubic meter, a density of 1.8 tons per cubic meter, the approved remaining time for disposal with 19 year (Ableidinger et al., 2007), and the actual Cu mass in bottom ash with 990 kg/yr. The critical flow for exported air-pollution-control (APC) residues into an underground storage facility is set equal to the actual flow. This assessment is justified, because the Viennese disposal practice meets the Swiss practice. The critical flow into soil is taken as equal to the Cu uptake through plants, which has been calculated for green and agricultural areas in Vienna (Kral et al., 2014). The critical flow into receiving water is based on environmental standards for surface waters with 9.3 microgram Cu per liter (μg Cu/l) (Bundesrepublik Österreich, 2006). There are two flows in total: the actual overflow from mixed sewer system is 37.5 million tons of water per year (Leitner, 2013). The actual Cu concentration of effluents from WTTP is 8.8 μg Cu/l (Kroiss et al., 2008).

(i) Copper is relevant from both a resource use and environmental impact viewpoint. On the one hand, Cu is essential for modern lifestyles, resulting in Cu waste fractions that have to be disposed of. From 1900 to 2000, about 0.7% of the Swiss Cu stock have been annually discarded in landfills (Wittmer, 2006). On the other hand, Cu is emitted from point and non-point sources and poses a risk for aquatic life. In Germany, it has been estimated that about 30% of total Cu loadings in receiving waters originate from urban areas (Böhm et al., 2001). In Vienna, Cu concentrations in sewage sludge are significantly larger than in rural areas (Kroiss et al., 2008), and Cu concentrations in urban soils are higher than in surrounding rural areas (Pfleiderer, 2011).
(ii) The Sankey-Diagram in Fig. 6a represents the annual Cu flows for the city of Vienna for the year 2008. Details about the SFA study have been published by (Kral et al., 2014). Calculating the sum of actual flows yields 1129 t Cu/yr (Fig. 6b). Thereof, 97.3% are disposed of in a local landfill, 1.5% are shipped to a foreign underground storage facility, 0.8% are deposited on urban soil, and 0.4% are entering receiving waters.

#### 2.3.2 Pb in Vienna

**Fig. 7:**
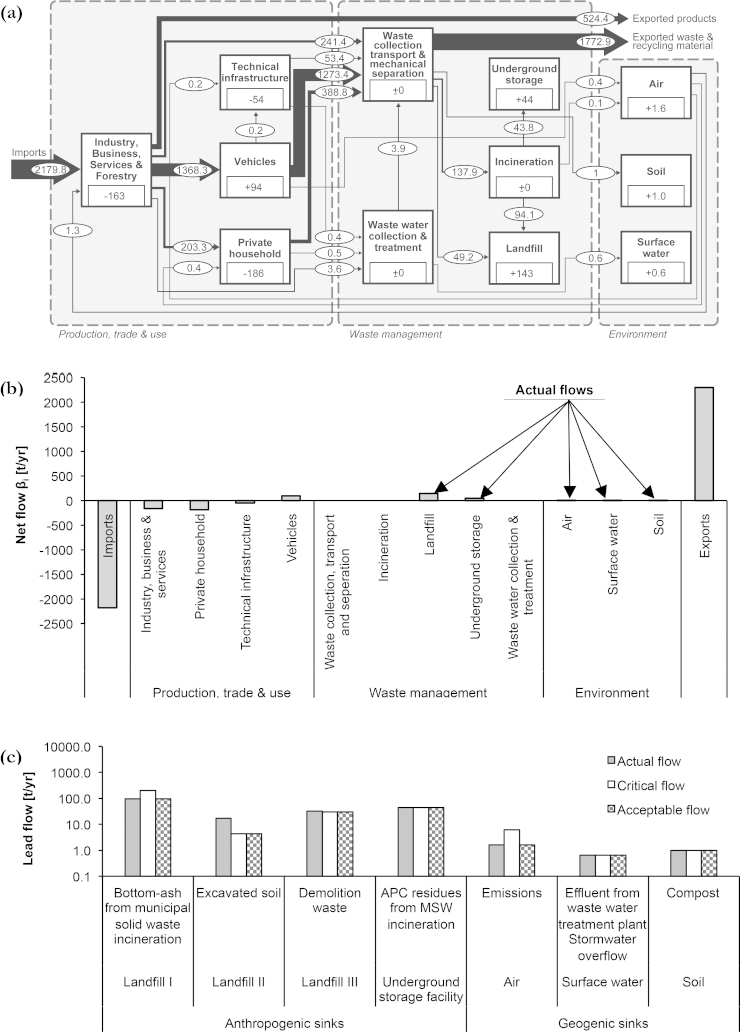
(a) Sankey-Diagram for lead in Vienna for the year 2008. Annual flow rates and changes in stocks are given in tons per year (t/yr), for stocks in tons (t). The flows are represented as Sankey diagrams proportional to the flow rate; figures for stocks are given within the process boxes. Deviations from mass balance are due to rounding. (b) The Source-Sink-Diagram presents the net flows *β_i_* for each process *i*. The actual flows are positive net flows into processes within the waste management sector and the environment. (c) The plot shows the results from normative assessment, namely the rated flows into sinks.
(iii) To determine the critical flows, we use (a) *damage criteria* for flows into geogenic sinks and (b) *proxy criteria* for flows into anthropogenic sinks.

(i) Human health is directly affected by emissions of Pb and Pb compounds. In Austria, Pb emissions to air decreased from 218 t/yr in the year 1990 to 13 t/yr in 2009 (Anderl et al., 2011). In 1993, lead has been banned from the Austrian petrol market. It is still used in accumulators, building coatings, tires and paints. Due to former Pb depositions and present diffusive losses, anthropogenic Pb is found in urban soils (Kreiner, 2004). Hence, it can be transferred to fodder and food, and may affect human health (WHO, 2007). Up to now, the Pb content in Austrian soils lacks of systematic nation-wide monitoring (Umweltbundesamt, 2010).
(ii) The Sankey-Diagram in Fig. 7a represents the annual Pb flows for the city of Vienna for the year 2008. Details about the SFA are given in the supplemental information. Calculating the sum of actual flows yields the sum of actual flows with 191 t Pb/yr, of which 75.4% entered a local landfill, 22.9% entered a foreign underground storage facility, 0.8% entered ambient air, 0.5% entered urban soil, and 0.3% entered receiving waters (Fig. 7b).

ad (a) The impact of flows into geogenic sinks is assessed in view of human health risks. Therefore, we apply the risk assessment model CalTOX 4.0 beta (McKone and Enoch, 2002). CalTOX has been developed to assess human exposures from continuous emissions to multiple environmental media. Background datasets can be found in the supplemental information. The method quantifies two *damage criteria*, namely the RL and the HI. To calculate the critical flows, we varied the actual flows as long as the *damage criteria* result acceptable risks. To demonstrate the method, widely used acceptable risks of 10^−6^ for RL and 1 for HI are selected, without discussing further acceptable risks (Kelly, 1991). Each actual flow is varied in a single scenario. Each variation results in two critical flows. One meets the acceptable RL, another meets the acceptable HI. Accordingly, three actual flows result in six scenarios. We picked out a stringent scenario, representing the minimum ratio between the critical flow and the actual flow.
ad (b) Flows into anthropogenic sinks are determined with *proxy criteria* in accordance to the Cu case study. The critical flow into landfills takes into account three different flows: Bottom ash from incineration, excavated soil, and demolition waste. An average annual flow is calculated with respect to landfill capacity. Therefore, the approved remaining landfill volume is divided by the approved disposal time (Ableidinger et al., 2007). The flow into the foreign underground storage facility is assessed in accordance with ES method. Therefore, the actual flow equals the critical flow. This approach has been chosen because the Viennese disposal practice is equal to the Swiss practice.

#### 2.3.3 PFOS in Switzerland

**Fig. 8:**
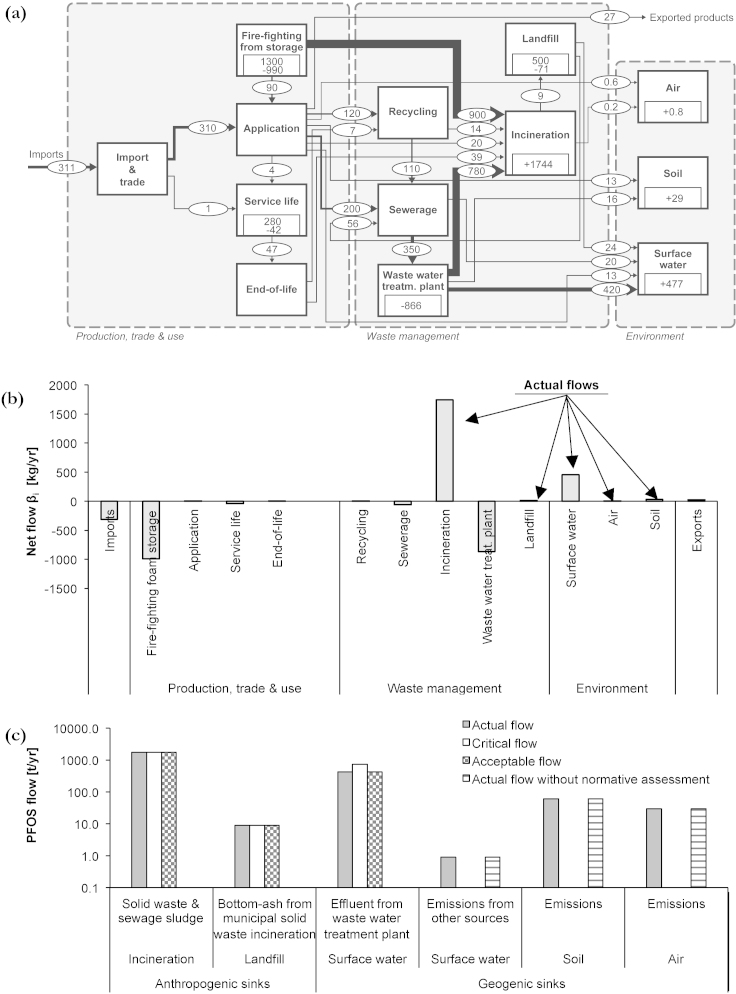
(a) Sankey-Diagram for PFOS in Switzerland for the year 2007. Annual flow rates and changes in stocks are given in kilogram per year (kg/yr), for stocks in kilogram (kg). The flows are represented as Sankey arrows proportional to the flow rate; figures for stocks are given within the process boxes. Deviations from mass balance are due to rounding. (b) The Source-Sink-Diagram presents the net flows *β_i_* for each process *i*. The actual flows are positive net flows into processes within the waste management sector and the environment. (c) The plot shows the results from normative assessment, namely the rated flows into sinks.
(iii) To determine the critical flows, we classified the actual PFOS flows into incineration as acceptable flow. This is in accordance to the EU Regulation, because PFOS is mineralized if it undergoes thermal treatment. Beyond the mineralized fraction, there is a very small PFOS fraction in incineration ashes. Due to the large concentrations of heavy metals and certain organic refractory substances, the ashes are classified as hazardous waste and are deposited in landfills or foreign underground storage facilities. According to ES method, this actual flow is set equal to the critical flow. This assessment is justified, because the Viennese disposal practice meets the Swiss practice. For flows into geogenic sinks, there is a lack of normative criteria. To estimate the critical flow from WWTP into surface waters, national standards are actually missing but under development (Götz et al., 2011). We used the critical concentration of 200 ng/l PFOS. This value is a provisional health advisory for drinking water, published by U.S. EPA (2009). Due to the lack of regulations and standards, other flows to the environment cannot be assessed and thus are excluded from normative assessment.

(i) Perfluorooctane sulfuric acid and its derivatives, collectively named PFOS, are persistent, bio-accumulative and toxic substances. They are regulated under the Persistent Organic Pollutants Regulation 850/2004 (European Parliament, 2004) and Regulation 2006/122/EG (European Parliament, 2006). In 2010, PFOS has been added to the convention with some exemptions for specific applications (European Commission, 2010). Hence, the European Union urges member states to implement strategies for careful PFOS management.
(ii) In Switzerland, the Federal Office of the Environment conducted a national study regarding the determination of stockpiles and waste fractions containing PFOS for the year 2007 (Buser and Morf, 2009). The corresponding Sankey-Diagram can be found in Fig. 8a. The production and use phase as well as – in consequence of – the waste water treatment plant (WWTP) act as main PFOS sources. Waste management provides the “anthropogenic sink” incineration, which mineralizes PFOS into carbon dioxide, water and HF. Environment provides the geogenic sinks hydrosphere, soil and atmosphere. Calculating the sum of actual flows yields 2260 kg PFOS/yr, of which 77.1% entered incineration, 0.4% entered landfills, and 22.5% entered geogenic sinks within hydrosphere, atmosphere and soil (Fig. 8b). The flow from WWTP to hydrosphere is determined with a concentration of 114 × 10^−9^ g PFOS per liter (ng/l) (Götz et al., 2011).

## 3 Results

The following sections give an overview of the results, and discuss the composition of the indicator score *λ* for each case study.

### 3.1 Overview

**Fig. 9:**
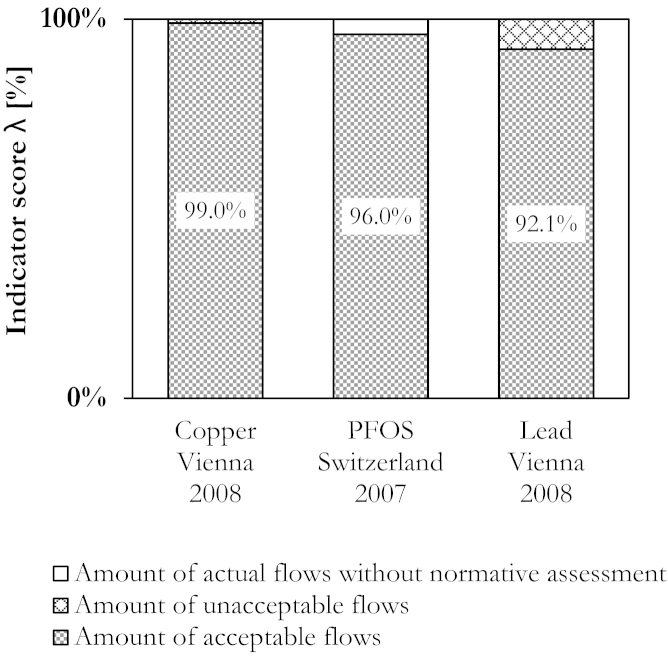
Compilation of indicator scores *λ* for the three case studies.

The indicator score *λ* for each case study is visualized in Fig. 9. It quantifies the share of acceptable flows in entire flows into sinks. The Cu metabolism in Vienna is limited by flows into the geogenic sink urban soil and receiving waters. The PFOS metabolism in Switzerland partly lacks of normative assessment due to lack of data, and the Pb metabolism in Vienna is restrained by flows into anthropogenic sinks such as local landfills. To compute the indicator score, the sum of acceptable and actual flows is needed and compiled in Table 4.

**Table 4:** Substance flow data required for calculating the indicator score.

Case study	Sink	Flow	Actual flow *F*_*i*_ (t/yr)	Critical flow *F*_c,*i*_ (t/yr)	Acceptable flow *F*_a,*i*_ (t/yr)
Cu	Landfill	Bottom-ash from MSW incineration	1097.2	2075.5	1097.2
	Underground storage facility	APC residues from MSW incineration (exported filter cake)	16.4	16.4	16.4
	Surface water	Stormwater overflow from mixed sewer system	3.9	0.3	0.3
	Surface water	Effluent from WWTP	1.6	1.7	1.6
	Soil	Deposition, fertilizer, pesticides, compost	8.8	1.1	1.1
		**Sum**	*F* = 1127.9	–	*F*_a_ = 1116.6

Pb	Landfill I	Bottom-ash from MSW incineration	94.7	198.5	94.7
	Landfill II	Excavated soil	17.3	4.3	4.3
	Landfill III	Demolition waste	31.9	29.8	29.8
	Underground storage facility	APC residues from MSW incineration (exported filter cake)	43.9	43.9	43.9
	Air	Emissions	1.6	6.1	1.6
	Surface water	Stormwater overflow from mixed sewer system Runoff from separated sewer system Effluent from WWTP	0.6	0.6	0.6
	Soil	Compost	1.0	1.0	1.0
		**Sum**	*F* = 190.9	–	*F*_a_ = 175.8

PFOS	Incineration	Solid waste and sewage sludge	1.744	1.744	1.744
	Landfill	Bottom-ash from MSW incineration	0.009	0.009	0.009
	Surface water	Effluent from WWTP	0.420	0.737	0.420
	Surface water	Emissions from other sources	0.060	n.a.	n.a.
	Soil	Emissions	0.029	n.a.	n.a.
	Air	Emissions	0.001	n.a.	n.a.
		**Sum**	*F* = 2.263	–	*F*_a_ = 2.173

*Notes*: –: not relevant; n.a.: not available due to a lack of normative criteria; APC: air pollution control; WWTP: waste water treatment plant; t/yr: tons per year, *F*_a_: sum of acceptable flows; *F*: sum of actual flows; Cu: copper, Pb: lead; PFOS: perfluorooctanesulfonate.

### 3.2 Cu in Vienna

99.0% of all actual Cu flows into sinks are acceptable. The sink “soil” poses a constraint for 0.7% of all actual Cu flows only. The sink “surface water” poses a constraint for 0.3% of all actual Cu flows. In detail, the following results have been obtained (Fig. 6c), whereas the percentage numbers refer to the sum of actual Cu flows (100% ≙ 1.128 t/yr):

• Landfill: 97.3% of flows are due to bottom-ash from MSW incineration. This is in compliance with landfill regulation. Calculated by the approved volume and service time of the landfill, the critical flow is 189% larger than the actual flow. Consequently, there is no constraint for the disposal of bottom-ash until the end of the approved service time. However, recycling is one option to disburden the landfill. If the stakeholders strive to increase the recycling rate of bottom-ash, the indicator calculation has to include a *criteria* with respect to both, the flow into the landfill and the recycled material flow (see Section 2.2.4).
• Underground storage facility: 1.5% of flows are due to APC residues from MSW incineration. These residues are exported and disposed of in approved underground storage facilities.
• Water: 0.1% of flows are from effluents from WWTP, fulfilling quality standards for surface waters. However, the actual flow is only 6% below the critical flow. Besides WWTP effluents, 0.3% of flows are within storm water overflow from mixed sewer systems to receiving waters. Applying the same standards as for effluents shows that the actual flow is 11 times larger than the critical flow. From an impact point of view, the flow complies with the quality standards in the receiving water. For the future, the indicator score might increase due to ongoing measures for reducing Cu bypassing waste water treatment via storm water overflow. Retention reservoirs and collection sewers are constructed at present in order to direct more urban surface waters including diffusive Cu losses toward WWTP (e.g. Stadt Wien, 2013).
• Soil: 0.1% of flows are acceptable, and 0.7% of flows are unacceptable because they exceed the critical level by a factor of eight. Cu flows into the soil are larger than the removal by plants. If no Cu leaching from soil is assumed (cf. ES methodology), Cu will be accumulating in specific soil compartments. The determination of the critical flow was rather crude due to a lack of accurate local data. Consequently, the quantity and quality of data has to be improved. This finding is in agreement with the recommendation of the Austrian Environmental Agency for a nation-wide soil metal monitoring program (Umweltbundesamt, 2010). Improved sampling with respect to horizontal and vertical sample collection and including the identification of potential hot spots would allow decreasing the uncertainty when determining the amount of unacceptable flows to the soil.

### 3.3 Pb in Vienna

92.1% of all actual Pb flows into sinks are acceptable. The sink “landfill” poses a constraint for 7.9% of all actual Pb flows. In detail, the following results have been obtained (Fig. 7c), whereas the percentage numbers refer to the sum of actual Pb flows (100% ≙ 191 t/yr):

• Landfills: 49.6% of flows are due to bottom-ash from MSW incineration. Due to the permit of the landfill, the critical flow is twice as large as the actual flow. 9.1% of all actual Pb flows originates from excavated soil to local landfills. If the landfill capacity should be fully utilized at the end of approved disposal time, only 2.3% of all actual Pb flows (instead of 9.1%) can be disposed of. The same pattern was found for Pb in demolition waste. 16.7% of Pb origins from demolition waste to landfills. If the landfill capacity should be utilized at the end of approved disposal time, only 15.6% of all actual Pb flows (instead of 16.7%) can be disposed of. If the disposal practice continues, landfills for excavated soil and for demolition waste will exceed their approved landfill volume within the approved time for disposal. In other words, the disposal of actual flows is in accordance with legal limits, but the disposal practice faces constraints. To overcome these constraints, landfill permissions have to be extended in time. Alternatively, waste fractions can be recycled complying with advanced quality standards (BRV, 2009), or wastes are directed to remote landfills beyond the system limits, which increases transport distances and costs.
• Underground storage: 22.9% of flows originate from APC residues from MSW incineration. The fractions are acceptable and exported into foreign underground storage facilities.
• Air, soil, water: 0.8% of flows enter ambient air, 0.5% enters urban soil, and 0.3% enters the water. The actual flows yield acceptable risks (RL: 7 × 10^−6^, HI: 0.26). Increasing the actual flow into air by a factor of three results in a critical flow (HI: 1). Even though the results yield acceptable human health risks, it has to be noted that the method is based on a uniform approach without spatial resolution. Thus, accidental hot spots representing possible risks for the local population are not included by this approach.

### 3.4 PFOS in Switzerland

96.0% of all actual PFOS flows into sinks are acceptable. 4% of all actual PFOS flows lack of normative assessment due to insufficient knowledge. In detail, the following results have been obtained (Fig. 8c), whereas the percentage numbers refer to the sum of actual PFOS flows (100% ≙ 2.260 t/yr):

• Incineration: 77.1% of PFOS flows are originating from solid waste and sewage sludge and are treated by MSW incineration. This flow is mineralized and is classified as acceptable flow, which is accordance to the EU Regulation (European Commission, 2010).
• Landfill: 0.4% of flows derives from residues of waste incineration and enters landfills in an acceptable manner. Today's PFOS emissions from landfills result from former rather than from present waste disposal. They have been assessed too.
• Water: Two flows enter the aquatic sphere. 18.6% of PFOS are within WWTP effluents and fulfill provisional drinking water standards (U.S. EPA, 2009). Up to now in Switzerland, legal limits are missing but under development (Götz et al., 2011). Even though standards will be available in the future, the monitoring of PFOS is rather challenging and expensive (e.g. Becker et al., 2008, Schultz et al., 2005). 2.7% of PFOS originate from additional sources. This flow has not been assessed, because data and standards were missing.
• Soil and air: 1.3% of PFOS enter the soil, and 0.04% enter the air. These flows, together with the 2.7% of flows into water have not been assessed. They pose potential threats on human and ecological health without a clear understanding about the fate and effects.

## 4 Conclusions

A new methodology is presented to assess if sinks are a constraint for waste and emission flows. The methodology is based on an indicator, ranging from 0% to 100%, and representing the ratio between the amount of environmentally acceptable and unacceptable flows into sinks. To our knowledge, it is the first indicator that indicates possible constraints for regional waste and emission flows to sinks in a region-wide perspective. The methodology is tested by three case studies on Cu and Pb in Vienna, and PFOS in Switzerland. The findings have several implications for material, environmental and waste management: (i) as long as the indicator score stays below 100%, there are unacceptable substance flows to geogenic and/or anthropogenic sinks. The information gained while determining the new indicator is highly instrumental for developing strategies and measures to decrease these flows and to raise the score up to a maximum value of 100%. (ii) The study shows the important part waste management (incineration and landfilling) plays as a relevant and necessary sink for anthropogenic material flows. (iii) For the three substances taken into account by the case studies, there are fractions (roughly 1–10%) that flow to inappropriate sinks. Still, these flows can pose an environmental problem and should be further investigated.

## 5 Outlook

The article starts with a statement, highlighting the constraints for anthropogenic carbon flows not only at the supply but also at the disposal side. In the case of carbon, the United Nations Framework Convention on Climate Change (UNFCCC) aims at managing the carbon content of the atmosphere. So, nations periodically update and publish national inventories of anthropogenic carbon emissions and removals by sinks. In the future, the systematic provision of source/sink inventories beyond carbon facilitates informed decisions about substance flow management in a comprehensive manner. This supports a sustainable resource management strategy with respect to safe sinks as both, need and constraint for waste and emissions.
