# Supplementary material for: Sinks as limited resources? A new indicator for evaluating anthropogenic material flows
Source: Ecol Indic. 2014 Nov;46:596–609. doi: 10.1016/j.ecolind.2014.06.027 (PMC4183748; doi:10.1016/j.ecolind.2014.06.027)
Supplement: Supplementary file 1 [file mmc1.docx]

**Supplement information for:**

**Kral, U, P.H. Brunner, P.C. Chen, S.R. Chen (2013). Sinks as limited resources? - A new indicator for evaluating anthropogenic material flows. Ecological Indicators**

**Summary**

This supplement information includes datasets for the case study about lead in Vienna. It comprises the scope, the descriptive and normative assessment of lead flows. Figure 1 highlights the framework to calculate the indicator score and the content of the supplement information in grey colored boxes.

Figure 1: Framework to calculate the indicator score. Tasks documented within the supplement information are colored grey, including the section numbers. Tasks documented within the supplement information are dark grey colored.

**Content**

[1 Scope 2](#_Toc377712127)

[2 Descriptive assessment 2](#_Toc377712128)

[2.1 Model development 2](#_Toc377712129)

[2.2 Model equations and data acquisition 4](#_Toc377712130)

[2.3 Balance equations 10](#_Toc377712131)

[3 Normative assessment 11](#_Toc377712132)

[3.1 Selection of criteria 11](#_Toc377712133)

[3.2 Risk assessment 11](#_Toc377712134)

[3.2.1 Approach 11](#_Toc377712135)

[3.2.2 Data acquisition 11](#_Toc377712136)

[4 Results 13](#_Toc377712137)

[4.1 Sankey-Diagram 13](#_Toc377712138)

[4.2 Actual risks 14](#_Toc377712139)

[4.3 Critical flows 15](#_Toc377712140)

[5 References 16](#_Toc377712141)

# Scope

Lead is selected as substance of interest. The system boundary in space is set with the administrative city limits. The system boundary in time is set with the year 2008.

# Descriptive assessment

In the sections that follow, the calculation of actual lead flows is documented. First, the model development includes the identification of processes and flows. Second, the model equations are defined. Third, the flows are balanced using the software STAN.

## Model development

The aim is to present a generic Pb flow model that allows the visualization of actual flows. This requires an understanding of the key Pb flows in the city of Vienna. The determination of processes and flows is based on previous studies, literature investigations, reports by local municipalities, and expert interviews. The generic Pb flow model can be found in Figure 2 and Figure 3. The process description is in accordance with [Kral et al. (2013](#_ENREF_17)) and can be found in Table 1.


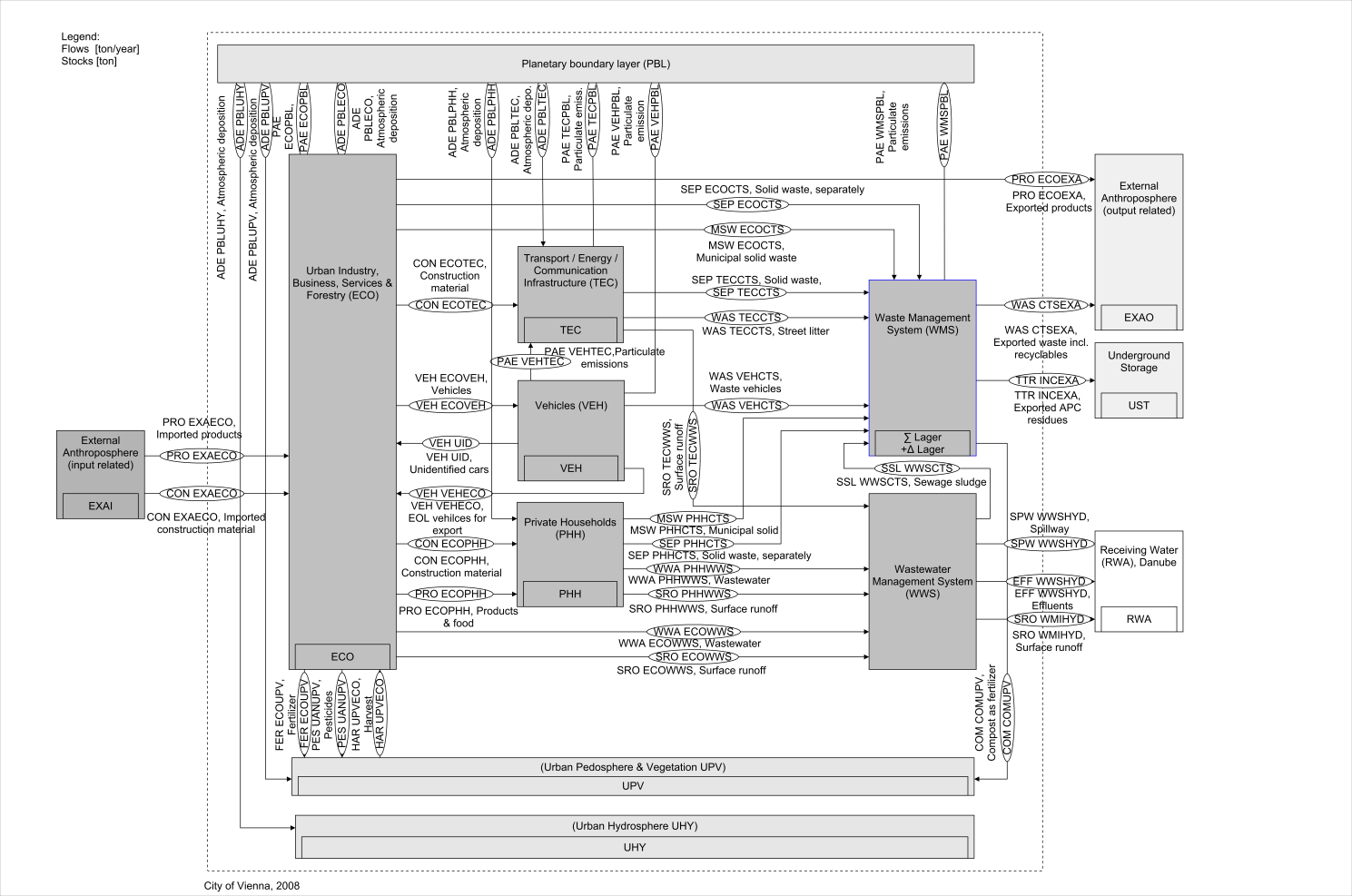


Figure 2: Generic lead flow model on top level. It covers 9 city internal processes of which 6 represent mainly anthropogenic activities (dark grey boxes) and 3 stand for environmental media (light grey boxes). Exterior processes are splitted in the supply and export as well as receiving waters in the hinterland. Regarding nomenclature, the flow acronyms refer to the type of flow (first three letters), to the source process (second three letters and to the sink process (last three letters). The stock acronyms refer to the type of stock only.


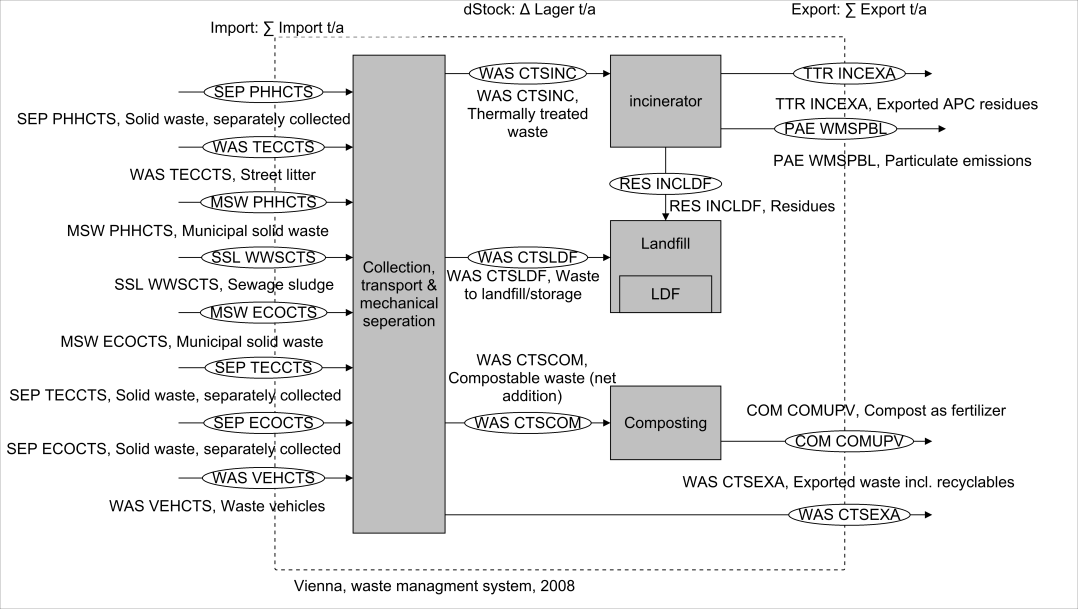


Figure 3: Generic lead flow model on 2^nd^ level. It disaggregates the “Waste Management System”.

Table 1: Process characterization.

| Process name | Characterization |
| --- | --- |
| External anthroposphere | Stands for the anthropogenic hinterland of the city. It delivers products and construction material to the city and receives exported products, waste and recyclables. |
| Industry, business, services and forestry | Covers economic activities as well as related buildings. Economic activities refer to the trade of goods, material processing and distribution for final consumption. The buildings are addressed for stock calculation including construction material and installations. |
| Transport, energy, and communication infrastructure | Covers immobile infrastructure and corresponding lead stock in transport networks, power grids and telecommunication networks. |
| Vehicles | Covers the mobile lead stocks such in cars, lorries, bikes, busses, trams, and trains. |
| Private Households | Covers anthropogenic activities of daily life and related buildings. Anthropogenic activities refer to residing, nourishing, cleaning, and communication. Related buildings like flats and houses are used for stock calculation of construction material and installations. |
| Waste Management System | Covers the collection, treatment and disposal of solid waste. The process is disaggregated which gives further insights into fluxes in view of incineration, composting and landfilling. |
| Waste Water Management System | Covers the collection and treatment of waste water. Material stocks are not taken into account. |
| Underground storage | External salt mines out of use act as final storage for hazardous residues from incineration. |
| Planetary boundary layer | Stands for the lowest part of the atmosphere that is influenced by its contact with the earth surface, usually several hundred meters high. |
| Urban pedosphere and vegetation | Consisting of urban soil and vegetation in parks, green areas and agricultural fields. |
| Urban hydrosphere | Urban water bodies, mainly rivers, groundwater, ponds, and small lakes. |
| Receiving waters | The hydrosphere that takes up both, waste water treatment effluents and combined sewer overflow from the city, such as the river Danube. |

## Model equations and data acquisition

This section provides a compilation of lead flow calculations. Table 2 includes the model equations and the flow results, Table 3 includes all input parameters.

Table 2: Model Equations and flow results

| **N°** | **Flow Acronym** | **Description** | **Equation** | **Mean Value [t/a]** | **Standard deviation**  **[t/a]** |
| --- | --- | --- | --- | --- | --- |
| 01 | ADE PBLECO | Atmospheric Deposition ECO | (Dep_wet_dry – Dep_wet) * A_ECO_ | 0,17 | 0,08 |
| 02 | ADE PBLPHH | Atmospheric Deposition PHH | (Dep_wet_dry – Dep_wet)* A_PHH_ | 0,39 | 0,19 |
| 03 | ADE PBLTEC | Atmospheric Deposition TEC | (Dep_wet_dry – Dep_wet) * A_TEC_ | 0,23 | 0,11 |
| 04 | ADE PBLUHY | Atmospheric Deposition UHY | (Dep_wet_dry – Dep_wet) * A_UHY_ | 0,07 | 0,04 |
| 05 | ADE PBLUPV | Atmospheric deposition UPV | (Dep_wet_dry – Dep_wet) * A_UPV_ | 0,73 | 0,35 |
| 06 | COM ANTUPV | Compost as fertilizer | m_comp_ * w_comp_ * c_comp_ | 0,97 | 1,08 |
| 07 | EFF WWSHYD | Effluents | m_Pb, inflow_ * **TC_WWTP_** | 0,05 | 0,01 |
| 08 | MSW ECOCTS | Municipal solid waste | (m_MSW_total_ -m_MSW_PHH_)***** c_MSW_ | 70,64 | 16,39 |
| 09 | MSW PHHCTS | Municipal solid waste | Cap_VIE_ *m_MSW_PHH_ * c_MSW_ | 66,59 | 15,45 |
| 10 | PAE ECOPBL | Particulate emissions | Calculated based on mass balance principle | 1,13 | 0,41 |
| 11 | PAE VEHPBL | Particulate emission  Low Duty Vehicles (LDV)  High Duty Vehicles (HDV) | LDV+HDV  LDV=mil_LDV * (e_LDV_brake_ * c_brake_ + e_LDV_tire_ * c_tire_) * **TC_VEH_**  HDV=mil_HDV * (e_HDV_brake_ * c_brake_ + e_HDV_tire_ * c_tire_) * **TC_VEH_** | 0,40 | 0,12 |
| 12 | PAE VEHTEC | Particulate emissions  Low Duty Vehicles (LDV)  High Duty Vehicles (HDV) | LDV+HDV  LDV=mil_LDV * (e_LDV_brake_ * c_brake_ + e_LDV_tire_ * c_tire_) * **TC_VEH_**  HDV=mil_HDV * (e_HDV_brake_ * c_brake_ + e_HDV_tire_ * c_tire_) * **TC_VEH_** | 0,23 | 0,05 |
| 13 | PAE WMSPBL | Particulate emissions | **m_waste_** * **e_waste_** | 0,06 | 0,01 |
| 14 | PRO ECOEXA | Exported products  End-of-life vehicles for export (VEH VEHECO)  Unidentified cars (VEH UID)  Lead and commodities out of lead (LC_exp_) | VEH VEHECO + VEH UID + LC_exp_  VEH VEHECO= car_dereg_exp_ * m_battery_ * Proxy_car_  VEH UID=(car_dereg_ + car_imp_reused_ – car_rereg_ – car_shred_ – car_dereg_exp_) * m_battery_ * Proxy_car_  LC_exp_= m_goods_exp_ * c_goods_ | 524,38 | 203,83 |
| 15 | PRO ECOPHH | Products & food  Electric & electronic appliances (EEA_PHH_)  Building material for roof and facades (BUI)  Food (FO) | EEA_PHH_ + BUI + FO  EEA_PHH_=**m_EEA_PHH_ECO_ * c_EEA_**  * Proxy_cap_ * Proxy_area_  BUI=( m_goods_imp_ – m_goods_exp_) * c_goods_ * Proxy_BUI_  FO= **m_food_*** **c_food_** | 203,33 | 91,81 |
| 16 | PRO EXAECO | Imported products  Batteries (BAT) = VEH ECOVEH  Electric & electronic appliances (EEA_total_)  Lead and commodities out of lead (LC_imp_)  Energy carriers (EC)  Food (FO) | BAT+EEA+LC+EC+FO  BAT=(car_reg_ * Proxy_car_ + car_stock_ / lifetime_battery_) * m_battery_  EEA_total_= **(m_EEA_PHH_ECO_ +m_EEA_ECO_)* c_EEA_**  * Proxy_cap_  LC_imp_= m_goods_imp_ * c_goods_  EC=**d_energy_** * **cv_energy_** * **c_energy_**  FO= **m_food_*** **c_food_** | 2179,76 | 313,74 |
| 17 | RES INCLDF | Bottom ash | m_MSW_total_ * c_MWS_ * **TC_INC_** | 94,69 | 21,98 |
| 18 | SEP ECOCTS | Solid waste, separate collected  Excavated soil (EXS)  Demolition waste (DEM)  PVC products (PVC)  Lead scrap (SCR)  Concrete waste (CON)  Waste electronics and equipment (WEE)  Lead mud (MUD) | EXS + DEM  EXS= m_soil_ * c_soil_ * RC_soil_ * Proxy_soil_  DEM=m_dem_ * c_dem_ * RC_dem_ * Proxy_dem_  PVC=(m_window_ * c_window_ + m_pipes_ * c_pipes_ + m_cables_ * c_cables_) * **Proxy_PVC_**  SCR=m_scrap_ * c_scrap_ * (1-Proxy_area_)  CON=m_con_ * c_con_ * (1-Proxy_area_)  WEE= **m_WEE *_ c_EEA *_ (1-**Proxy_area_)  MUD=m_mud_ * c_mud_ | 170,90 | 45,07 |
| 19 | SEP PHHCTS | Solid waste, separate collected  Excavated soil (EXS)  Demolition waste (DEM)  PVC products (PVC)  Lead scrap (SCR)  Concrete waste (CON)  Waste electronics and equipment (WEE) | EXS + DEM  EXS= m_soil_ * c_soil_ * RC_soil_ * Proxy_soil_  DEM=m_dem_ * c_dem_ * RC_dem_ * Proxy_dem_  PVC=(m_window_ * c_window_ + m_pipes_ * c_pipes_ + m_cables_ * c_cables_) * **Proxy_PVC_**  SCR=m_scrap_ * c_scrap_ * Proxy_area_  CON=m_con_ * c_con_ * Proxy_area_  WEE= **m_WEE *_ c_EEA *_** Proxy_area_ | 322,61 | 97,60 |
| 20 | SEP TECCTS | Solid waste, separate collected  Excavated soil (EXS)  Demolition waste (DEM) | EXS + DEM  EXS= m_soil_ * c_soil_ * RC_soil_ * Proxy_soil_  DEM=m_dem_ * c_dem_ * RC_dem_ * Proxy_dem_ | 53,36 | 18,57 |
| 21 | SPW WWSHYD | Spillway | (SRO TECWWS + SRO PHHWWS + SRO ECOWWS) * Ratio_SST_ * Ratio_overflow_ | 0,40 | 0,04 |
| 22 | SRO ECOWWS | Surface runoff (ADE PBLECO) | (Dep_wet_dry – Dep_wet) * A_ECO_ | 0,39 | 0,19 |
| 23 | SRO PHHWWS | Surface runoff (ADE PBLPHH) | (Dep_wet_dry – Dep_wet)* A_PHH_ | 0,23 | 0,11 |
| 24 | SRO TECWWS | Surface runoff  Atmospheric depositions (ADE PBLTEC)  Particulate emissions Low Duty Vehicles (LDV)  Particulate emissions High Duty Vehicles (HDV)  Street litter (WAS TECCTS) | ADE PBLTEC + LDV + HDV – WAS TECCTS  ADE PBLTEC=(Dep_wet_dry – Dep_wet) * A_TEC_  LDV=mil_LDV * (e_LDV_brake_ * c_brake_ + e_LDV_tire_ * c_tire_) * **TC_VEH_**  HDV=mil_HDV * (e_HDV_brake_ * c_brake_ + e_HDV_tire_ * c_tire_) * **TC_VEH_**  WAS TECCTS=(ADE PBLTEC + LDV + HDV) * Ratio_streetlitter_ | 0,42 | 0,06 |
| 25 | SRO WMIHYD | Surface runoff | (SRO ECOWWS + SRO PHHWWS + SRO TECWWS) * (1-Ratio_SST_) | 0,19 | 0,02 |
| 26 | SSL WWSCTS | Sewage sludge | m_Pb, inflow_ * **TC_WWTP_** | 3,75 | 0,87 |
| 27 | TTR INCEXA | Exported APC residues | m_MSW_total_ * c_MWS_ * **TC_INC_** | 43,91 | 9,88 |
| 28 | VEH ECOVEH | Vehicles | (car_reg_ * Proxy_car_ + car_stock_ / lifetime_battery_) * m_battery_ | 1804,65 | 291,13 |
| 29 | VEH UID | Unidentified cars | (car_dereg_ + car_imp_reused_ – car_rereg_ – car_shred_ – car_dereg_exp_) * m_battery_ * Proxy_car_ | 361,52 | 190,24 |
| 30 | VEH VEHECO | EOL vehicles for export | car_dereg_exp_ * m_battery_ * Proxy_car_ | 74,84 | 39,38 |
| 31 | WAS CTSCOM | Compostable waste (net addition) | m_comp_ * w_comp_ * c_comp_ | 0,97 | 1,08 |
| 32 | WAS CTSEXA | Exported waste incl. recyclables  Excavated soil (EXS)  Demolition waste (DEM)  Concrete waste (CON)  Waste vehicles (WAS VEHCTS)  Lead scrap (SCR) | EXS + DEM + CON +  EXS=m_soil_ * c_soil_ * (1-RC_soil_)  DEM=m_dem_ * c_dem_ * (1-RC_dem_)  CON=m_con_ * c_con_  WAS VEHCTS= car_shred_ * m_battery_ + car_stock_ / lifetime_battery_ * m_battery_  SCR= m_scrap_ * c_scrap_ | 1698,12 | 1369,46 |
| 33 | WAS CTSINC | Thermal treated waste | m_MSW_total_ * c_MWS_ | 137,23 | 22,53 |
| 34 | WAS CTSLDF | Waste to landfill/storage  Excavated soil (EXS)  Demolition waste (DEM) | EXS + DEM  EXS= m_soil_ * c_soil_ * (1-RC_soil_)  DEM=m_dem_ * c_dem_ * (1-RC_dem_) | 49,14 | 20,41 |
| 35 | WAS TECCTS | Street litter | See line 24 | 0,05 | 0,01 |
| 36 | WAS VEHCTS | Waste vehicles  Batteries from shredded cars (SRE)  Exported batteries due to maintenance (MAI) | SRE + MAI  SRE=car_shred_ * m_battery_  MAI= car_stock_ / lifetime_battery_ * m_battery_ | 1347,74 | 1365,32 |
| 37 | WWA ECOWWS | Wastewater (unknown sources) | m_Pb, inflow_ + SPW WWSHYD – WWA PHHWWS - (SRO TECWWS + SRO PHHWWS + SRO ECOWWS) * Ratio_SST_ | 3,37 | 0,88 |
| 38 | WWA PHHWWS | Wastewater | m_water_ * c_water_ | 0,32 | 0,06 |

Table 3: List of input parameters (non bold=single values, bold=matrices)

| **Input**  **Parameter** | **Description of data** | **Unit** | **Value** | **Uncertainty level** | **Reference** |
| --- | --- | --- | --- | --- | --- |
| Cap_AUT_ | Inhabitants Austria | # | 8,347,341 | 1 | ([Statistik Austria, 2012](#_ENREF_33)) |
| Cap_VIE_ | Inhabitants Vienna | # | 1,674,909 | 1 | ([Lebhart, 2010](#_ENREF_21)) |
| Dep_wet_dry | Deposition rate (wet & dry) | g Pb/(ha*yr) | 44.60 | 3 | ([Spiegel, 2003](#_ENREF_27)) |
| Dep_dry | Deposition rate (wet) | g Pb/(ha*yr) | 7.20 | 3 | ([Kalina et al., 2000](#_ENREF_15)) |
| A | Total City Area | ha | 41,487 | 1 | ([Lebhart, 2010](#_ENREF_21)) |
| A_UHY_ | Urban Hydrosphere (Open Water Bodies) | ha | 1,933 | 1 | ([Lebhart, 2010](#_ENREF_21)) |
| A_TEC_ | Traffic Surface | ha | 5,981 | 1 | ([Lebhart, 2010](#_ENREF_21)) |
| A_PHH_ | Residential Area | ha | 10,267 | 1 | ([Lebhart, 2010](#_ENREF_21)) |
| A_ECO_ | Public Facilities and production areas | ha | 4,381 | 1 | ([Lebhart, 2010](#_ENREF_21)) |
| A_UPV_ | Green Space Area | ha | 18,925 | 1 | ([Lebhart, 2010](#_ENREF_21)) |
| Proxy_cap_ | Ratio of inhabitants Vienna/Austria | - | 0.201 | - | - |
| Proxy_area_ | Area Ratio of residential land use in contrast to total built area = A_PHH_ /(A_PHH_+A_ECO_) | - | 0.701 | - | - |
| **m_EEA_PHH_ECO_** | National sale of electric & electronic appliances (household appliances, consumed in PHH and ECO) | kg | ∑=32,436,269 | 2 | ([EAK, 2009](#_ENREF_7)) |
| **m_EEA_ECO_** | National sale of electric & electronic appliances (commercial appliances) | kg | ∑=1,624,894 | 2 | ([EAK, 2009](#_ENREF_7)) |
| **c_EEA_** | Average lead concentration in electric & electronic appliances | % | 0-1.6 | 3 | ([EMPA, 2009](#_ENREF_8)) |
| m_battery_ | Lead content per car battery | kg Pb/# | 13 | 3 | assumption |
| **m_food_** | Food imports to Vienna | t | 1,236 | 1 | ([Magistratsabteilung 05, 2011](#_ENREF_24)) |
| **C_food_** | Lead concentration in different food types | mg Pb/kg food | 0.013-0.263 | 2 | ([USDA, 2011](#_ENREF_38)) |
| **m_goods_imp_** | Imported “Lead and commodities out of lead” | t/yr | 136 | 3 | ([Magistratsabteilung 05, 2011](#_ENREF_24)) |
| **m_goods_exp_** | Exported “Lead and commodities out of lead” | t/yr | 98 | 3 | ([Magistratsabteilung 05, 2011](#_ENREF_24)) |
| Proxy_BUI_ | Amount of lead that is consumed the building sector PHH in relation to the net difference of imported and exported “Lead and commodities out of lead” | % | 15 | 3 | ([Forum Nachhaltiges Bauen, 2013](#_ENREF_10)) |
| **c_goods_** | Lead concentration for goods within the category “Lead and commodities out of lead” | % | 89.9 | 3 | ([Reisinger et al., 2009](#_ENREF_26)) |
| **d_energy_** | Final energy demand in Vienna, categorized into various energy carriers. | TJ | ∑=1,259,22 | 1 |  |
| **cv_energy_** | Colorific values of various energy carriers | TJ/t | 0.015-0.063 | - |  |
| **c_energy_** | Lead concentration in energy carriers | mg/kg |  | 3 |  |
| car_reg_ | New registered cars in Austria | # | 293,697 | - | ([Statistik Austria, 2009b](#_ENREF_32)) |
| car_dereg_ | Deregistered Cars in Austria | # | 993,354 |  | Calculated based on mass balance principle |
| car_imp_reused_ | Imported second hand cars in Austria | # | 28,696 |  | ([Wirtschaftskammer Österreich, 2012](#_ENREF_44)) |
| car_rereg_ | Re-Registered Cars in Austria | # | 738,690 |  | ([Statistik Austria, 2009a](#_ENREF_31)) |
| car_dereg_exp_ | Deregistered and exported cars in Austria | # | 37,629 |  | ([Wirtschaftskammer Österreich, 2012](#_ENREF_44)) |
| Car_Shred_ | Shredded cars in Austria | # | 63,975 |  | ([BMLFUW, 2011a](#_ENREF_4)) |
| Proxy_car_ | Proxy representing Viennese and Austrian car ownership | % | 0.1534 | - |  |
| car_stock_ | Number of registered cars in Vienna | # | 657,192 | 1 | ([Statistik Austria, 2008](#_ENREF_30)) |
| lifetime_battery_ | Lifetime of a battery | Years | 7 | 3 | assumption |
| m_MSW_total_ | Mixed waste from households and similar institutions | t/yr | 579,888 | 2 | ([Wiener Umweltschutzabteilung MA22, 2011](#_ENREF_42)) |
| m_MSW_PHH_ | Mixed waste from private households, national average | kg/cap/yr | 168 | - | ([BMLFUW, 2011a](#_ENREF_4)) |
| c_MSW_ | Lead concentration in mixed waste | mg/kg | geomittel(200;280) | 2 | ([Morf and Taverna, 2006](#_ENREF_25); [Taverna et al., 2011](#_ENREF_34)) |
| m_soil_ | Amount of excavated soil | t/yr | 2,978,726.89 | 1 | ([Wiener Umweltschutzabteilung MA22, 2011](#_ENREF_42)) |
| c_soil_ | Lead concentration in excavated soil | mg/kg | 16.1 | 3 | ([Woisetschlaeger et al., 2000](#_ENREF_45)) |
| RC_soil_ | Recycling quote for excavated soil | % | 64 | 3 | ([BMLFUW, 2011b](#_ENREF_5)) |
| Proxy_soil_ | Allocation proxy for soil to PHH, ECO and TEC | - | 1/3 | 2 | assumption |
| m_dem_ | Amount of demolition waste | t/yr | 1,090,976 | 1 | ([Wiener Umweltschutzabteilung MA22, 2011](#_ENREF_42)) |
| c_dem_ | Lead concentration in demolition waste | mg/kg | 85 | 3 | ([König, 2006](#_ENREF_16)) |
| RC_dem_ | Recycling quote for demolition waste | % | 65.6 | 3 | ([BMLFUW, 2011a](#_ENREF_4)) |
| Proxy_dem_ | Allocation proxy for demolition waste to PHH, ECO and TEC | - | 1/3 | 2 | Assumption |
| m_window_ | Amount of window frames | t/yr | geomittel(2,000;4000) | 1 | ([BMLFUW, 2002a](#_ENREF_2)) |
| c_window_ | Lead concentration in window frames | % | 0.7 | 2 | ([AgPU, 2004](#_ENREF_1)) |
| m_pipes_ | Amount of pipes | t/yr | geomittel(1,000;1000) | 1 | ([BMLFUW, 2002a](#_ENREF_2)) |
| c_pipes_ | Lead concentration in pipes | % | 2.7 | 2 | ([AgPU, 2004](#_ENREF_1)) |
| m_cables_ | Amount of cables | t/yr | geomittel(4,000;8000) | 1 | ([BMLFUW, 2002a](#_ENREF_2)) |
| c_cables_ | Lead concentration in cables | % | 2.0 | 2 | ([AgPU, 2004](#_ENREF_1)) |
| **Proxy_PVC_** | Allocation proxy for PVC waste fractions to PHH and ECO | % | various | 3 | ([BMLFUW, 2002a](#_ENREF_2)) |
| m_scrap_ | Amount of lead scrap | t/yr | 263.57 | 2 | ([Wiener Umweltschutzabteilung MA22, 2011](#_ENREF_42)) |
| c_scrap_ | Lead concentration of lead scrap | % | 90 | 3 | ([König, 2006](#_ENREF_16)) |
| m_con_ | Amount of concrete waste | t/yr | 432,294.65 | 2 | ([Wiener Umweltschutzabteilung MA22, 2011](#_ENREF_42)) |
| c_con_ | Lead concentration in concrete waste | g/t | 50 | 3 | Assumed to be 50% of maximal listed Pb concentration ([VDZ, 1996](#_ENREF_39)) |
| **m_WEE_** | Collected WEEE in PHH of Vienna | t/a | 9,044 | 1 | ([EAK, 2009](#_ENREF_7)) |
| mil_LDV | Mileage of low duty vehicles in Vienna | Veh-km | 5,694,000,000 | 1 | ([Holzapfel and Riedel, 2011](#_ENREF_12)) |
| mil_HDV | Mileage of low duty vehicles in Vienna | Veh-km | 803,000,000 | 1 | ([Holzapfel and Riedel 2011](#_ENREF_28) |
| e_LDV_brake_ | Total wear rate of brake lining in low duty vehicles | mg /veh-km | 12.5 | 2 | ([Winther and Slento, 2010](#_ENREF_43)) |
| e_HDV_brake_ | Total wear rate of brake lining in low duty vehicles | mg /veh-km | 54.7 | 2 | ([Winther and Slento, 2010](#_ENREF_43)) |
| c_brake_LDV_ | Lead content of brake linings in low duty vehicles | mg Pb/kg brake pad | 11.381 mg | 2 | ([Westerlund, 2001](#_ENREF_41)) |
| c_brake_HDV_ | Lead content of brake linings in high duty vehicles | mg Pb/kg brake pad | geommittel(158;656) | 3.04 | ([Westerlund, 2001](#_ENREF_41)) |
| e_LDV_tire_ | Total wear rate of tires in low duty vehicles | mg/veh-km | 136.38 | 2 | ([Luhana et al., 2004](#_ENREF_23); [Winther and Slento, 2010](#_ENREF_43)) |
| e_HDV_tire_ | Total wear rate of tires in high duty vehicles | mg/veh-km | 136.38 | 2 | ([Luhana et al., 2004](#_ENREF_23); [Winther and Slento, 2010](#_ENREF_43)) |
| c_tire_LDV_ | Lead content of tires in low duty vehicles | mg/kg | 80.5 | 2 | ([Luhana et al., 2004](#_ENREF_23); [Winther and Slento, 2010](#_ENREF_43)) |
| c_tire_HDV_ | Lead content of tires in high duty vehicles | mg/kg | 80.5 | 2 | ([Luhana et al., 2004](#_ENREF_23); [Winther and Slento, 2010](#_ENREF_43)) |
| **TC_MOV_** | Transfer coefficients describing the ratio of particulate emissions entering individual sinks | % | 20; 31; 49 | 2 | ([Hulskotte et al., 2006](#_ENREF_13)) |
| m_mud_ | Amount of lead mud in Vienna | t/yr | 0.18 | 1 | ([Wiener Umweltschutzabteilung MA22, 2011](#_ENREF_42)) |
| c_mud_ | Lead concentration of lead mud | % | 50 | 3 | assumption |
| m_Pb, inflow_ | Leadinflow to Waste Water Treatment Plant | t Pb/yr | 4.09 | 2 | ([Kroiss et al., 2008](#_ENREF_18)) |
| **TC_WWTP_** | Transfer coefficients for lead in the Viennese waste water treatment plant | % | 1.3; 7.0; 91.7 | 2 | ([Kroiss et al., 2008](#_ENREF_18)) |
| **TC_INC_** | Transfer coefficients for lead in the Viennese incinerator plants | % | 1, 31; 69 | 2 | ([Taverna et al., 2011](#_ENREF_34)) |
| m_water_ | Revenue water | *m3/yr* | 122.775.000 | 1 | ([Tomenendal, 2011](#_ENREF_35)) |
| c_water_ | Lead concentration in Viennese drinking water | *µg/l* | 2.6 | 2 | ([Haider et al., 2002](#_ENREF_11)) |
| m_street_ | Amount of collected street litter | *t/yr* | 39,495 | 1 | ([Wiener Umweltschutzabteilung MA22, 2011](#_ENREF_42)) |
| m_comp_ | Applied compost on land | *t/yr* | 33,000 | 1 | ([Weinmar, 2011](#_ENREF_40)) |
| w_comp_ | Water content of compost | *%* | 41 | 3 | ([Umweltbundesamt, 2000](#_ENREF_36)) |
| c_comp_ | Lead concentration in compost | *mg Pb/kg* | 71.6 | 3 | ([Umweltbundesamt, 2000](#_ENREF_36)) |
| Ratio_overflow_ | Percentage of surface runoff ending up as stormwater overflow | *%* | 50 | - | ([Fenz, 1999](#_ENREF_9)) |
| Ratio_SST_ | Ratio surface water collected by mixed sewer system based on network lengths | *%* | 81 | - | ([I C Consultants Ltd, 2001](#_ENREF_14); [Lampert et al., 1997](#_ENREF_20); [Lehmann, 2011](#_ENREF_22)) |
| **m_waste_** | Mixed waste flows into municipal solid waste incinerators in Vienna | *t/yr* | ∑=508.254 | 1 | ([Kronberger, 2011](#_ENREF_19)) |
| **e_waste_** | Lead emission factors for municipal solid waste incinerators in Vienna | *g Pb/t waste* | 0.220; 0.054 | 2 | ([BMLFUW, 2002b](#_ENREF_3)) |
| Ratio_streetlitter_ | Ration of Pb in street litter in relation to Pb deposition on traffic surfaces | *%* | 10 | - | assumption |

## Balance equations

The quantified flows from Table 2 do not fulfill the mass balance on each process. Consequently, we balanced the flows by error propagation and data reconciliation. This step was carried out by the software STAN ([Cencic, 2012](#_ENREF_6)). The balanced flows are used to determine actual flows into sinks (Table 5).

Table 4: Unbalanced versus balanced flows in t/yr.

| Acronym | Flow name | Mass flow (unbalanced) | ± Mass flow (unbalanced) | Mass flow (balanced) | ± Mass flow (balanced) |
| --- | --- | --- | --- | --- | --- |
| ADE PBLECO | Atmospheric deposition | 0,17 | 0,03 | 0,17 | 0,03 |
| ADE PBLPHH | Atmospheric deposition | 0,39 | 0,07 | 0,39 | 0,07 |
| ADE PBLTEC | Atmospheric deposition | 0,23 | 0,04 | 0,23 | 0,04 |
| ADE PBLUHY | Atmospheric deposition | 0,07 | 0,01 | 0,07 | 0,01 |
| ADE PBLUPV | Atmospheric deposition | 0,73 | 0,12 | 0,73 | 0,10 |
| COM COMUPV | Compost as fertilizer | 0,97 | 1,08 | 0,97 | 0,76 |
| CON ECOPHH | Construction material | 0,00 | 0,00 | 0,00 | 0,00 |
| CON ECOTEC | Construction material | 0,00 | 0,00 | 0,00 | 0,00 |
| CON EXAECO | Imported construction material | 0,00 | 0,00 | 0,00 | 0,00 |
| EFF WWSHYD | Effluents | 0,05 | 0,01 | 0,05 | 0,01 |
| FER ECOUPV | Fertilizer | 0,00 | 0,00 | 0,00 | 0,00 |
| HAR UPVECO | Harvest | 0,00 | 0,00 | 0,00 | 0,00 |
| MSW ECOCTS | Municipal solid waste | 70,64 | 16,39 | 70,63 | 16,39 |
| MSW PHHCTS | Municipal solid waste | 66,59 | 15,45 | 66,58 | 15,45 |
| PAE ECOPBL | Particulate emissions | 1,13 | 0,08 | 1,13 | 0,07 |
| PAE TECPBL | Particulate emissions | 0,00 | 0,00 | 0,00 | 0,00 |
| PAE VEHPBL | Particulate emission | 0,40 | 0,12 | 0,40 | 0,10 |
| PAE VEHTEC | Particulate emissions | 0,24 | 0,05 | 0,24 | 0,05 |
| PAE WMSPBL | Particulate emissions | 0,06 | 0,01 | 0,06 | 0,01 |
| PES UANUPV | Pesticides | 0,00 | 0,00 | 0,00 | 0,00 |
| PRO ECOEXA | Exported products | 524,38 | 203,83 | 524,38 | 203,83 |
| PRO ECOPHH | Products & food | 203,33 | 91,81 | 203,33 | 91,81 |
| PRO EXAECO | Imported products | 2179,76 | 313,74 | 2179,76 | 313,74 |
| RES INCLDF | Residues | 94,69 | 21,98 | 94,06 | 16,39 |
| SEP ECOCTS | Solid waste, separate collected | 170,90 | 45,07 | 170,82 | 45,06 |
| SEP PHHCTS | Solid waste, separate collected | 322,61 | 97,60 | 322,23 | 97,48 |
| SEP TECCTS | Solid waste, separate collected | 53,36 | 18,57 | 53,35 | 18,57 |
| SPW WWSHYD | Spillway | 0,40 | 0,04 | 0,40 | 0,04 |
| SRO ECOWWS | Surface runoff | 0,39 | 0,07 | 0,39 | 0,07 |
| SRO PHHWWS | Surface runoff | 0,23 | 0,04 | 0,23 | 0,04 |
| SRO TECWWS | Surface runoff | 0,42 | 0,06 | 0,42 | 0,06 |
| SRO WMIHYD | Surface runoff | 0,19 | 0,02 | 0,19 | 0,02 |
| SSL WWSCTS | Sewage sludge | 3,75 | 0,87 | 3,92 | 0,62 |
| TTR INCEXA | Exported APC residues | 43,91 | 9,88 | 43,78 | 9,43 |
| VEH ECOVEH | Vehicles | 1804,65 | 291,13 | 1804,65 | 291,13 |
| VEH UID | Unidentified cars | 361,52 | 190,24 | 361,52 | 190,24 |
| VEH VEHECO | EOL vehicles for export | 74,84 | 39,38 | 74,84 | 39,38 |
| WAS CTSCOM | Compostable waste (net addition) | 0,97 | 1,08 | 0,97 | 0,76 |
| WAS CTSEXA | Exported waste incl. recyclables | 1698,12 | 1369,46 | 1772,92 | 968,58 |
| WAS CTSINC | Thermal treated waste | 137,23 | 22,53 | 137,91 | 16,46 |
| WAS CTSLDF | Waste to landfill/storage | 49,14 | 20,41 | 49,16 | 20,41 |
| WAS TECCTS | Street litter | 0,05 | 0,01 | 0,05 | 0,01 |
| WAS VEHCTS | Waste vehicles | 1347,74 | 1365,32 | 1273,39 | 968,56 |
| WWA ECOWWS | Wastewater | 3,37 | 0,88 | 3,20 | 0,62 |
| WWA PHHWWS | Wastewater | 0,32 | 0,06 | 0,32 | 0,06 |

# Normative assessment

The sections that follow include the selection of the criteria, the methodology to calculate the criteria, as well the data needed to apply the methodology.

## Selection of criteria

To assess the actual flows into natural sinks, two impact indices are selected in view of human health. The “risk level” stands for the carcinogenic risk and the “hazard-index” stands for the non-carcinogenic risk.

## Risk assessment

The sections that follow include a brief overview of the risk assessment approach and details about the background data.

### Approach

To compute the two *impact indices*, the software tool CalTOX 4.0 has been applied. The model has been developed by Lawrence Berkeley National Laboratory. CalTOX is designed to run in Microsoft Excel® and is available online([State of California, 2007](#_ENREF_29)). The CalTOX model structure is visualized in Figure 5.The approach covers three steps in total, though methodological details can be found in the user manual ([University of California, 1994](#_ENREF_37)):

- First, multimedia transport simulation is used to compute the fate of lead in the environment. The model turns out the result of substance flows and stocks in each compartment when the system reaches an equilibrium state.
- Second, exposure assessment defines the relation between the environmental medium and the exposure medium. The result is the “average daily dose rate” for each exposure route.
- Third, the *impact indices* were calculated with the dose-response relationship of lead.

Figure 4: CalTOX model structure (adopted from [State of California, 1993](#_ENREF_28); [State of California, 2007](#_ENREF_29)). It consist of (a) multimedia transport simulation, (b) exposure assessment, and (c) the risk algorithm that yields two impact indices.

### Data acquisition

In general, CalTOX requires four types of input data. The points that follow include the abbreviation from the default values, provided by the CalTOX spreadsheet.

1. Emission flows in mol/day. These data are provided by the SFA model. Therefore, 1,59 t Pb/yr enter air, 0,97 t Pb/yr enter soil and 0,64 t Pb enter water (sec. 2, Table 17).

Table 5: Anthropogenic flows for CalTOX model inputs

| **Flow short name** | **Flow name** | **Sink** | **Actual flow [ton/year]** | **Actual flow into sink**  **[ton/year]** | **Actual flow into sink^*^**  **[mol/day]** |
| --- | --- | --- | --- | --- | --- |
| PAE ECOPBL | Particulate emissions | Air | 1,12 | 1.59 | 21.02 |
| PAE VEHPBL | Particulate emission | Air | 0,41 |  |  |
| PAE WMSPBL | Particulate emissions | Air | 0,06 |  |  |
| COM COMUPV | Compost as fertilizer | Soil | 0,97 | 0.97 | 12.81 |
| EFF WWSHYD | Effluents | Water | 0,05 | 0.64 | 8.50 |
| SPW WWSHYD | Spillway | Water | 0,40 |  |  |
| SRO WMIHYD | Surface runoff | Water | 0,19 |  |  |

Note: *=Conversion factor from [ton/year] to [mol/day]: 10^6^[g/t] * 1mol/207.21 [g*t] * 1 [year/365days]

1. Chemical properties. In total 36 parameters are needed. Default values for Pb are provided in CalTOX and used to run the model.
2. 58 landscape properties. In total 58 parameters are needed. 11 out of 58 landscape parameters are adopted for Vienna (Table 18). 47 out of 58 parameters are default values, provided in CalTOX, and were used to run the model.
3. Exposure factors. In total 52 parameters are needed.21 out of 52 exposure factors are adopted for Vienna (Table 19). 31out of 52 exposure factors are default values, provided in CalTOX, and were used to run the model.

Table 6: Vienna's landscape properties in CalTOX

| **Landscape properties** | **Variable name** | **value** |
| --- | --- | --- |
| Contaminated area in m2 | Area | 414871000 |
| Annual average precipitation (m/d) | rain | 0.002 |
| Land surface runoff (m/d) | runoff | 0.0008 |
| Atmospheric dust load (kg/m3) | rhob_a | 2.39E-08 |
| Ground-water recharge (m/d) | recharge | 0.00003 |
| Thickness of the ground soil layer (m) | d_g | 0.01 |
| Soil particle density (kg/m3) | rhos_s | 1600 |
| Water content in surface soil (vol fraction) | beta_g | 0.15 |
| Fraction of land area in surface water | f_arw | 0.05 |
| Ambient environmental temperature (K) | Temp | 285 |
| Yearly average wind speed (m/d) | v_w | 319200 |

Table 7: Vienna's exposure properties in CalTOX

| **Human Exposure Factors** | **Variable name** | **value** |
| --- | --- | --- |
| Body weight (kg) | BW | 74 |
| Surface area (m2/kg) | SAb | 0.0254 |
| Fluid Intake (L/kg-d) | Ifl | 0.0208 |
| Fruit and vegetable intake (kg/kg-d) | Ifv | 0.0055 |
| Grain intake (kg/kg-d) | Ig | 0.0000 |
| Milk intake (kg/kg-d) | Imk | 0.0095 |
| Meat intake (kg/kg-d) | Imt | 0.0021 |
| Egg intake (kg/kg-d) | Iegg | 0.0004 |
| Fish intake (kg/kg-d) | Ifsh | 0.0002 |
| Soil ingestion (kg/d) | Isl | 0.0002 |
| Fraction of water needs from ground water | fw_gw | 0.0251 |
| Fraction of water needs from surface water | fw_sw | 0 |
| Frctn frts & vgtbls that are exposed produce | fabv_grd_v | 0.4 |
| Fraction of fruits and vegetables local | flocal_v | 0.364 |
| Fraction of grains local | flocal_g | 0.069 |
| Fraction of milk local | flocal_mk | 0 |
| Fraction of meat local | flocal_mt | 0 |
| Fraction of eggs local | flocal_egg | 0 |
| Fraction of fish local | flocal_fsh | 0 |
| Exposure duration (years) | ED | 30 |
| Averaging time (days) | AT | 28878.8 |

# Results

The sections that follow include the Sankey-Diagram for actual flows, the actual risk based on the actual flows, and the critical flows in view of acceptable risks.

## Sankey-Diagram


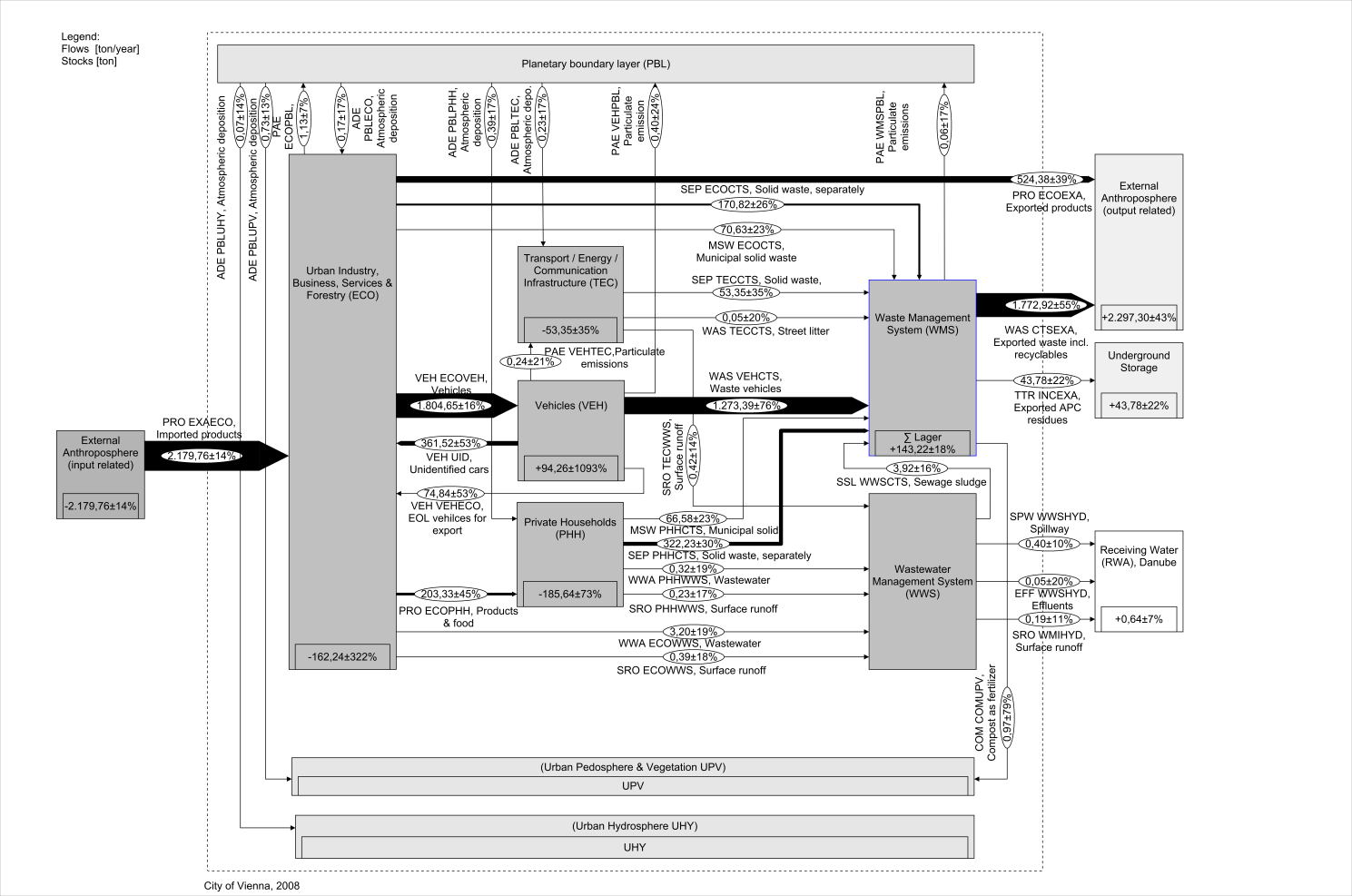


Figure 5: The Sankey diagram for lead in Vienna on an annual base for the year 2008. Flow rates and changes in stocks are given in mass/time, for stocks in mass. The flows are represented as Sankey arrows proportional to the flow rate; figures for stocks are given within the process boxes.


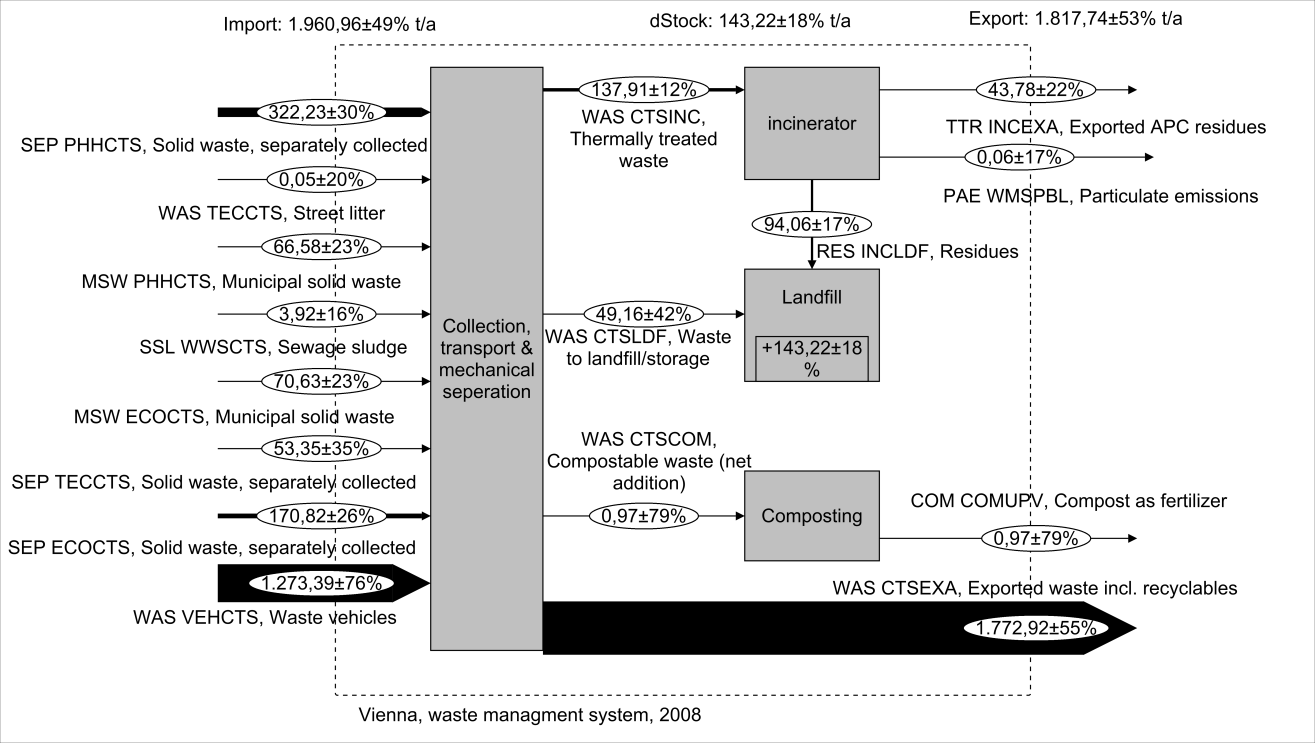


Figure 6:The Sankey diagram for lead in the Viennese waste management sector, on an annual base for the year 2008. Flow rates and changes in stocks are given in mass/time, for stocks in mass. The flows are represented as Sankey arrows proportional to the flow rate; figures for stocks are given within the process boxes.

## Actual risks

The actual flows into natural sinks yield a risk level of 7.41 E-8(Carcinogenic risk) and a hazard-index of 0.26 (non-carcinogenic risk). Table 20 and Table 21 shows that the risk level and the hazard-index are mainly driven by the ingestion of lead due to vegetables grown above the ground (exposed produce).

Table 8: The analysis of carcinogenic risk

| Exposure route | Risk | Contribution rate |
| --- | --- | --- |
| **Inhalation** | **1.25 E-8** | **16.82%** |
| **Ingestion** | **6.15 E-8** | **83.01%** |
| Water | 1.34 E-9 | 1.81% |
| Exposed produce | 6.01 E-8 | 81.03% |
| Unexposed produce | 2.54 E-14 | 0.00% |
| Meat | 0 | 0% |
| Milk | 0 | 0% |
| Eggs | 0 | 0% |
| Fish | 0 | 0% |
| Soil | 1.23 E-10 | 0.17% |
| **Dermal** | **1.33 E-10** | **0.18%** |
| Total Risk | 7.41 E-8 | 100% |

Table 9: The analysis of hazard-index

| Exposure route | Hazard-index | Contribution rate |
| --- | --- | --- |
| **Inhalation** | **1.83E-02** | **6.98%** |
| **Ingestion** | **2.43E-01** | **92.82%** |
| Water | 5.30E-03 | 2.03% |
| Exposed produce | 2.37E-01 | 90.61% |
| Unexposed produce | 1.00E-07 | 0.00% |
| Meat | 0 | 0% |
| Milk | 0 | 0% |
| Eggs | 0 | 0% |
| Fish | 0 | 0% |
| Soil | 4.87E-04 | 0.19% |
| **Dermal** | **5.24E-04** | **0.20%** |
| HQ | 2.62E-01 | 100% |

## Critical flows

Scenario 4 yields the minimum ratio between the critical flow and the actual flow (Table 22 and Table 23).

Table 10: Critical flows in view of the risk level.

| Scenario N° | Sink | Actual flow (tons/year) | Critical flow (tons/year) | Tolerable flow (tons/year) | Ratio critical / actual flow |
| --- | --- | --- | --- | --- | --- |
| 1 | Air | 1,59 | 21,93 | 20,34 | 14 |
|  | Water | 0,64 | 0,64 | 0,00 |  |
|  | Soil | 0,97 | 0,97 | 0,00 |  |
| 2 | Air | 1,59 | 1,59 | 0,00 |  |
|  | Water | 0,64 | 461,35 | 460,74 | 717 |
|  | Soil | 0,97 | 0,97 | 0,00 |  |
| 3 | Air | 1,59 | 1,59 | 0,00 |  |
|  | Water | 0,64 | 0,64 | 0,00 |  |
|  | Soil | 0,97 | 2.647,11 | 2.646,14 | 2.733 |

Table 11:Critical flows in view of the hazard-index.

|  | Sink | Actual flow (tons/year) | Critical flow (tons/year) | Tolerable flow (tons/year) | Ratio critical / actual flow |
| --- | --- | --- | --- | --- | --- |
| 4 | Air | 1.59 | 6.13 | 4.54 | 4 |
|  | Water | 0.64 | 0.64 | 0.00 |  |
|  | Soil | 0.97 | 0.97 | 0.00 |  |
| 5 | Air | 1.59 | 1.59 | 0.00 |  |
|  | Water | 0.64 | 90.76 | 90.15 | 141 |
|  | Soil | 0.97 | 0.97 | 0.00 |  |
| 6 | Air | 1.59 | 1.59 | 0.00 |  |
|  | Water | 0.64 | 0.64 | 0.00 |  |
|  | Soil | 0.97 | 605.05 | 604.08 | 625 |

# References

AgPU, 2004. Produktions-, Verbrauchs-und Abfalldaten für PVC in Deutschland unter Einbeziehung der Verwertung [Data about production, consumption and waste generation for PVC in Germany]. Arbeitsgemeinschaft PVC und Umwelt (AgPU), Bonn.

BMLFUW, 2002a. Behandlungs- und Verwertungswege für PVC-Abfälle [Treatment and disposal routes for PVC waste]. Wien.

BMLFUW, 2002b. Stand der Technik bei Abfallverbrennungsanlagen [State of the art of waste incineration plants]. Bundesministerium für Land- und Forstwirtschaft, Umwelt und Wasserwirtschaft (BMLFUW), Wien.

BMLFUW, 2011a. Bundes-Abfallwirtschaftsplan 2011 [Federal Waste Management Plan 2011]. Bundesministerium für Land- und Forstwirtschaft, Umwelt und Wasserwirtschaft (BMLFUW), Wien.

BMLFUW, 2011b. Bundes-Abfallwirtschaftsplan 2011 (Band 2) [Federal Waste Management Plan 2011 (part 2)]. Bundesministerium für Land- und Forstwirtschaft, Umwelt und Wasserwirtschaft (BMLFUW), Wien.

Cencic, O., 2012. Software platform STAN (short for subSTance flow ANalysis). Url: [www.stan2web.net](http://www.stan2web.net), Access date: 28th September 2012. Vienna Universtiy of Technology, Vienna.

EAK, 2009. Tätigkeitsbericht 2008 [Annual Report 2008]. Elektroaltgeräte Koordinierungsstelle Austria GmbH (EAK), Wien.

EMPA, 2009. Material composition. Url: <http://ewasteguide.info/material_composition>, Access date: 25th August 2012. Eidgenössische Materialprüfanstalt, Dübendorf.

Fenz, R., 1999. Gewässerschutz bei Entlastungsbauwerken der Mischkanalisation [Water protection of buildings regarding combined sewer systems]. PhD thesis, Technische Universität Wien, Wien.

Forum Nachhaltiges Bauen, 2013. Blei - Ökobilanz [Lead - Life cycle assessment]. Url: <http://www.nachhaltiges-bauen.de/baustoffe/Blei>, Access date: 1st October 2013. Forum Nachhaltiges Bauen.

Haider, T., Haider, M., Wruss, W., Sommer, R., Kundi, M., 2002. Lead in drinking water of Vienna in comparison to other European countries and accordance with recent guidelines. International Journal of Hygiene and Environmental Health 205, 399-403.

Holzapfel, P., Riedel, R., 2011. Verkehrsmodell Wien [Traffic model Vienna]. Personal communication, reicipent: Kellner, K., retrieved on 4th December 2011.

Hulskotte, J.H.J., Schaap, M., Visschedijk, A., 2006. Brake wear from vehicles as an important source of diffuse copper pollution. Conference: 10th Int. Specialised Conference on Diffuse Pollution ans Sustainable Basin Management, 18th - 22nd September 2006, Instanbul.

I C Consultants Ltd, 2001. Pollutants in Urban Waste Water and Sewage Sludge. European Communities, Luxembourg.

Kalina, M., Leder, K., Kramer, S., Puxbaum, H., 2000. Nasse Deposition im Land Wien Oktober 99 - September 00 [Wet deposition in Vienna, Oktober 99 - September 00]. Technische Univeristät Wien. Institut für Analytische Chemie. Abteilung für Umweltanalytik, Wien.

König, S., 2006. Stoffflussbasiertes Verfahren zur Bestimmung von Metallfrachten in Abfällen [Methods to determine metal flows in waste]. Master thesis, Technische Universität Wien, Wien.

Kral, U., Lin, C.-Y., Kellner, K., Ma, H.-w., Brunner, P.H., 2013. The copper balance of cities: Exploratory insights into a European and an Asian city. Journal of Industrial Ecology. in press.

Kroiss, H., Morf, L.S., Lampert, C., Zessner, M., 2008. Optimiertes Stoffflussmonitoring für die Abwasserentsorgung Wiens [Optimized substance flow monitoring of the waste water treatment plant in Vienna]. Technische Universität Wien, Wien.

Kronberger, R., 2011. Solid waste Inputs to Viennese incinerators 2008. Personal communication, reicipent: Kral, U., retrieved on 21st November 2011, Wien.

Lampert, C., Stark, W., Kernbeis, R., Brunner, P.H., 1997. Stofffflussanalyse der Siedlungsentwässerung der beiden Regionen "Gresten" und "Grafenwörth", Studie im Rahmen

des Niederösterreichischen Klärschlammkonzeptes [Susbtance flow analysis of regional water discharge]. Technische Universität Wien, Wien.

Lebhart, G., 2010. Statistisches Jahrbuch der Stadt Wien 2010 [Statistical Yearbook of Vienna 2010]. Magistrat der Stadt Wien, Magistratsabteilung 5 - Finanzwesen, Wien.

Lehmann, T., 2011. Wastewater input into Viennas waste water system, ratio of mixed/separated sewer system in Vienna. Personal communication, reicipent: Kellner, K., retrieved on 20th July 2011, Wien.

Luhana, L., Sokhi, R., Warner, L., Mao, H., Boulter, P., 2004. Characterisation of Exhaust Particulate Emissions from Road Vehicles (PARTICULATES). Deliverable 8: Measurement of non-exhaust particulate matter. Hatfield, UK.

Magistratsabteilung 05, 2011. Aussenhandelsstatistik für Wien 2008 [Trade Statistic for Vienna 2008, restricted for public access]. Personal communication, reicipent: Kral, U., retrieved on 5th March 2012, Wien.

Morf, L.S., Taverna, R., 2006. Monitoringkonzept zur Ermittlung von Ursachen für Veränderungen der Schwermetallgehalte im Wiener Restmüll [Monitoring concept to investigate the change in heavy metal concentrations in residual waste of Vienna]. GEO Partner AG Umweltmanagement, Zürich.

Reisinger, H., Jakl, T., Quint, R., Schöller, G., Müller, B., Riss, A., Brunner, P.H., 2009. Ressourcenpotenzial und Umweltbelastung der Schwermetalle Blei, Cadmium und Quecksilber in Österreich. in: Jakl, T. (Ed.), CHEM-NEWS XVIII. Bundesministerium für Land- und Forstwirtschaft, Umwelt und Wasserwirtschaft, Wien, pp. 56-64.

Spiegel, H., 2003. Atmospheric deposition of heavy metals onto arable land in Austria. Conference: Concerted Action AROMIS, 24th - 25th November 2003, Kloster Banz, Germany.

State of California, 1993. CalTOX, A Multimedia Total Exposure Model For Hazardous-Waste Sites. Part I: Executive Summary. California.

State of California, 2007. CalTOX Download Instructions. Url: [www.dtsc.ca.gov/AssessingRisk/ctox_dwn.cfm](http://www.dtsc.ca.gov/AssessingRisk/ctox_dwn.cfm), Access date: 25th March 2012. California.

Statistik Austria, 2008. Kraftfahrzeuge - Bestand [Number of registered vehicles]. Url: [www.statistik.at/web_de/statistiken/verkehr/strasse/kraftfahrzeuge_-_bestand/index.html](http://www.statistik.at/web_de/statistiken/verkehr/strasse/kraftfahrzeuge_-_bestand/index.html), Access date: 3rd August 2011. Wien.

Statistik Austria, 2009a. Kfz-Gebrauchtzulassungen 2008 [Number of re-registred cars 2008]. Url: [www.statistik.at](http://www.statistik.at), Access date: 1st March 2012. Wien.

Statistik Austria, 2009b. Kfz-Neuzulassungen Jänner bis Dezember 2008 [Number of new registered vehicles]. Url: [www.statistik.at](http://www.statistik.at), Access date: 8th February 2012. Wien.

Statistik Austria, 2012. Bevölkerung Österreichs 2001-2050 nach Haushaltsgröße [Austrian Inhabitants 2001-2050]. Url: [www.statistik.at/web_de/statistiken/bevoelkerung/demographische_prognosen/haushalts_und_familienprognosen/023535.html](http://www.statistik.at/web_de/statistiken/bevoelkerung/demographische_prognosen/haushalts_und_familienprognosen/023535.html), Access date: 1st May 2012. Wien.

Taverna, R., Rolland, C., Böker, C., Kirchner, A., 2011. Routinemäßiges Stoffflussmonitoring auf der MVA Spittelau - Messperiode 1.5.09-30.4.10 [Routine monitoring measaures of the incinerator Spittelau in Vienna - monitoring period: 1.5.09-30.4.10]. GEO Partner AG, Magistratsabteilungen 22 und 48 der Stadt Wien, Fernwärme Wien GmbH, Zürich.

Tomenendal, A., 2011. Freshwater Supply Vienna. Personal communication, reicipent: Kellner, K., retrieved on 19th July 2011.

Umweltbundesamt, 2000. Qualität von Komposten aus der getrennten Sammlung – Ergebnisse der Kompostanalysen [Qualtity of compost from seperated collection]. Umweltbundesamt Österreich, Wien.

University of California, 1994. CalTOX™, A Multimedia Total Exposure Model For Hazardous-Waste Sites - Spreadsheet User’s Guide. California.

USDA, 2011. National Nutrient Database. Url: [www.nal.usda.gov/fnic/foodcomp/search/](http://www.nal.usda.gov/fnic/foodcomp/search/), Access date: 25th November 2011. U.S. Department of Agriculture (USDA).

VDZ, 1996. Umweltverträglichkeit von Zement und Beton [Environmental impact of cement and concrete]. Verein Deutscher Zementwerke (VDZ), Düsseldorf.

Weinmar, K., 2011. Kompostproduktion und -verbrauch in Wien [Compost production and consumption in Vienna]. Personal communication, reicipent: Kellner, K., retrieved on 2nd August 2010.

Westerlund, K.G., 2001. Metal emissions from Stockholm traffic - wear of brake linings. Stockholm.

Wiener Umweltschutzabteilung MA22, 2011. Abfallstatistik Wien 2008 [Waste statistic for Vienna 2008]. Personal communication, reicipent: Kellner, K., retrieved on 30th June 2011, Wien.

Winther, M., Slento, E., 2010. Heavy metal emissions for danish road transport. NERI Technical Report no. 780. Aarhus University, Aarhus, Denmark.

Wirtschaftskammer Österreich, 2012. Rahmendaten - Importe und Exporte gebrauchter Fahrzeuge [Data about imports and exports of second life cars]. Url: <http://wko.at/fahrzeuge/main_frame/statistik/JB/Seite5.11%202012.pdf>, Access date: 9th August 2012.

Woisetschlaeger, G., Musser, S., Lindlbauer, A., Wruss, W., 2000. Bestandsaufnahme der Bodenbelastung in Wien PAK, KW, Schwermetalle [Assessement of soil contamination in Vienna]. Magistratsabeilung 22, Wien.
